# Prevention and control of cholera with household and community water, sanitation and hygiene (WASH) interventions: A scoping review of current international guidelines

**DOI:** 10.1371/journal.pone.0226549

**Published:** 2020-01-08

**Authors:** Lauren D’Mello-Guyett, Karin Gallandat, Rafael Van den Bergh, Dawn Taylor, Gregory Bulit, Dominique Legros, Peter Maes, Francesco Checchi, Oliver Cumming

**Affiliations:** 1 Department of Disease Control, Faculty of Infectious and Tropical Diseases, London School of Hygiene and Tropical Medicine, London, United Kingdom; 2 Environmental Health Unit, Médecins Sans Frontières, Brussels, Belgium; 3 LuxOR, Luxembourg Operational Research Unit, Médecins Sans Frontières, Luxembourg; 4 Public Health Unit, Médecins Sans Frontières, Amsterdam, Netherlands; 5 Water, Sanitation and Hygiene, UNICEF, New York, New York, United States of America; 6 Global Task Force on Cholera Control, World Health Organization, Geneva, Switzerland; 7 Department of Infectious Disease Epidemiology, Faculty of Epidemiology and Population Health, London School of Hygiene and Tropical Medicine, London, United Kingdom; Johns Hopkins Bloomberg School of Public Health, UNITED STATES

## Abstract

**Introduction:**

Cholera remains a frequent cause of outbreaks globally, particularly in areas with inadequate water, sanitation and hygiene (WASH) services. Cholera is spread through faecal-oral routes, and studies demonstrate that ingestion of *Vibrio cholerae* occurs from consuming contaminated food and water, contact with cholera cases and transmission from contaminated environmental point sources. WASH guidelines recommending interventions for the prevention and control of cholera are numerous and vary considerably in their recommendations. To date, there has been no review of practice guidelines used in cholera prevention and control programmes.

**Methods:**

We systematically searched international agency websites to identify WASH intervention guidelines used in cholera programmes in endemic and epidemic settings. Recommendations listed in the guidelines were extracted, categorised and analysed. Analysis was based on consistency, concordance and recommendations were classified on the basis of whether the interventions targeted within-household or community-level transmission.

**Results:**

Eight international guidelines were included in this review: three by non-governmental organisations (NGOs), one from a non-profit organisation (NPO), three from multilateral organisations and one from a research institution. There were 95 distinct recommendations identified, and concordance among guidelines was poor to fair. All categories of WASH interventions were featured in the guidelines. The majority of recommendations targeted community-level transmission (45%), 35% targeted within-household transmission and 20% both.

**Conclusions:**

Recent evidence suggests that interventions for effective cholera control and response to epidemics should focus on case-centred approaches and within-household transmission. Guidelines did consistently propose interventions targeting transmission within households. However, the majority of recommendations listed in guidelines targeted community-level transmission and tended to be more focused on preventing contamination of the environment by cases or recurrent outbreaks, and the level of service required to interrupt community-level transmission was often not specified. The guidelines in current use were varied and interpretation may be difficult when conflicting recommendations are provided. Future editions of guidelines should reflect on the inclusion of evidence-based approaches, cholera transmission models and resource-efficient strategies.

## Introduction

Cholera remains a major public health threat in many parts of the world [1], particularly in areas facing complex emergencies [2–4]. Cholera outbreaks generally occur when water, sanitation and hygiene (WASH) services are inadequate or compromised [3, 5–14], and cholera remains a leading cause of disease outbreaks globally [15–17], with an increasing rate and intensity [18]. Originating in the Indian Subcontinent, cholera spread beyond the Ganges delta in 1817, and the current and ongoing seventh pandemic of *Vibrio cholerae* El Tor began in 1961 [19]. Adjusting for incomplete reporting, some 2.9 million cholera cases (1.3–4.0 million uncertainty range) and 95,000 deaths (21,000–143,000 uncertainty range) are estimated to occur across 69 cholera-endemic countries annually [20]. Sub-Saharan Africa and South Asia account for the largest proportion of global cholera morbidity and mortality [18, 21], with many cities acting as transmission hotspots [21–24].

Diarrhoeal diseases such as cholera are transmitted through the faecal-oral route. Infection with *V*. *cholerae* can originate from a susceptible person ingesting the bacteria from environmental point sources (e.g. contaminated water in lakes and rivers, or a faecal-contaminated environment) [25]: this is known as the environment-to-human transmission pathway [26, 27]. Infection with *V*. *cholerae* can also occur between infected and susceptible individuals [28, 29], from consuming contaminated food [30–37] or water at the point of use (POU) [37–43] that has been contaminated by a cholera case or through caring for existing cholera cases, particularly among household contacts of a case [28]: this is known as the human-to-human transmission pathway. During outbreaks, recurrent environment-to-human reinfection of the population may also occur through ingestion of *V*. *cholerae* through contaminated environmental point sources, due to sustained contamination of the environment by symptomatic and asymptomatic cholera cases [25, 44, 45]. Both transmission pathways occur through the faecal-oral routes of diarrhoeal disease transmission commonly known as the F-diagram [46].

Transmission models that only include ingestion of *V*. *cholerae* through environmental point sources, or environment-to-human transmission, cannot explain the steep rise in case numbers usually seen in outbreaks [27, 45, 47]. Spatiotemporal analyses of cholera in endemic and epidemic settings have instead demonstrated clusters of cases within 200m distances of case-households during the first five days after index cases present with symptoms [48–50], and a 100-fold higher risk of household contacts of cases to contract the disease compared to those outside the household [43, 51–54]. Research on the genomics of cholera transmission has also demonstrated strong phylogenetic similarities among same-household cases [43, 55–58], and a recent paper found 80% of transmission occurs between people who share a household [55]. Accordingly, faecal-oral transmission of cholera within the household, predominantly through the human-to-human transmission pathway, may far better explain the propagated and explosive nature of cholera outbreaks than community-level transmission from exposure to environmental point sources and environment-to-human transmission [27, 29, 45, 59–62].

These relatively recent findings suggest that efforts to prevent and control cholera could benefit from focusing on the domains of transmission: within-household and community-level. Typically, cholera response measures for prevention and control have included a mix of WASH interventions, Oral Cholera Vaccination (OCV) and, in some cases, prophylactic antibiotics. Strategies that seek to control and contain cholera outbreaks in epidemic and endemic settings could implement these measures to the household–delivered through case-centred strategies (i.e. delivery of interventions to cases and their households or close contacts) or case area targeted interventions (CATIs) (i.e. delivery of interventions to a defined area surrounding cases) [47]–and take advantage of the natural clustering of cases within a given distance and effectively reduce within-household transmission [44, 49, 63]. Whereas strategies that seek to prevent cholera could implement community-level measures–potentially aligning resources with longer term WASH-related disease control efforts [64]–and effectively reduce environment-to-human transmission during outbreaks [65, 66] and prevent disease among populations deemed to be at an elevated risk of recurrent cholera [21]. Targeted approaches would also be efficient across resource-limited contexts, as part of a phased approach or in contrast to mass intervention campaigns [67].

There is currently global momentum to tackle cholera and an internationally agreed road map to eliminate the disease by 2030 [68]. While it is accepted that large scale investment in water and sanitation infrastructure in Europe and the Americas led to the elimination of cholera and a reduction in other diarrhoeal diseases [63, 66, 69–89], there is a paucity of evidence to support which WASH interventions are most relevant for cholera prevention and control in currently cholera-affected populations [70, 90]. Multiple WASH guidelines exist for cholera prevention and control in both endemic and epidemic settings. However, the guidelines used in low- and middle-income countries (LMICs) vary considerably between and within international organisations and it is unclear to what extent these guideline recommendations are predicated upon experience rather than published evidence. Whilst appropriate cholera responses will always be specific to the geographical and social context, it is important that these responses are informed by the best possible evidence and updated models of cholera transmission or, in the absence of rigorous evidence, a combination of theoretical reasoning, best operational judgement and documented practice, even if unpublished [91–93].

Given the above, we conducted a scoping review of current, international and accessible WASH guidelines for cholera prevention and control to analyse consistency and concordance among recommended interventions, and to assess how guidelines seek to prevent and control cholera whilst aligning with current conceptual models of cholera transmission, in order to make recommendations for their improvement.

## Methods

### Search strategy

The search strategy sought to identify all relevant international guidelines (published and in press) and was limited to English and French languages. The review is reported according to the Preferred Reporting Items for Systematic Reviews and Meta‐Analyses extension for Scoping Reviews (PRISMA-ScR) guidelines [94]. The review was not pre-registered prior to publication.

The websites of organisations who typically respond to cholera were searched, including the Global WASH Cluster (GWC), World Health Organization (WHO), United Nations Children’s Fund (UNICEF), United Nations High Commissioner for Refugees (UNHCR), International Organization of Migration (IOM), Médecins Sans Frontières (MSF), Oxfam, International Committee for the Red Cross and Red Crescent (ICRC), International Federation of the Red Cross and Red Crescent Societies (IFRC), Action Contre la Faim (ACF), Care International, Save The Children, Norwegian Refugee Council, the Sphere Project, United States Centers for Disease Control and Prevention (CDC) and International Centre for Diarrhoeal Disease Research Bangladesh (ICDDR’B).

Reference sections of guidelines were hand-searched for any additional relevant guidelines. Journal articles did not meet the inclusion criteria for this review and reference databases were not searched for guidelines. A full list of websites searched can be found in S1 Appendix. Prior to searching organisations’ websites for available guidelines, a research librarian assisted in the development of search terms and, in collaboration with the authors, provided advice on organisations where guidelines could be found. Search terms have been provided in S2 Appendix.

### Inclusion and exclusion criteria

Guidelines were eligible for inclusion if they were available after 1999 and up to and including January 2019, and recommended interventions for cholera prevention and control. Only household- and community-level WASH interventions were included. Any guideline in which interventions were proposed for high-, middle- or low-income countries was included in the review.

Guidelines for infection prevention and control (IPC) or WASH in Cholera Treatment Centres or Units (CTCs or CTUs) or Health Care Facilities (HCFs) were excluded as these will be addressed in a separate review. Guidelines published in languages other than English or French, guidelines for non-human subjects or for other water-related or outbreak-prone diseases were excluded. Historical versions of guidelines that have been subsequently updated, and have been assumed by the authors to be no longer in use, and country-specific guidelines were also excluded from the review.

### Data extraction and analysis

All retrieved documents were transferred to Endnote X9 (Clarivate Analytics, Boston, USA) and de-duplicated. Records were screened according to the inclusion criteria described. Data were extracted by two reviewers (LDG and KG) and cross-checked for accuracy. Any disagreement between reviewers was resolved through discussion and consensus. Data were extracted into an MS Excel (Microsoft, Redmond, VA, USA) sheet for each of the guidelines on the following: agency/author and year of publication, overall content of the guideline and whether the guideline proposed interventions for urban, rural, endemic and/or epidemic contexts.

**Fig 1:**
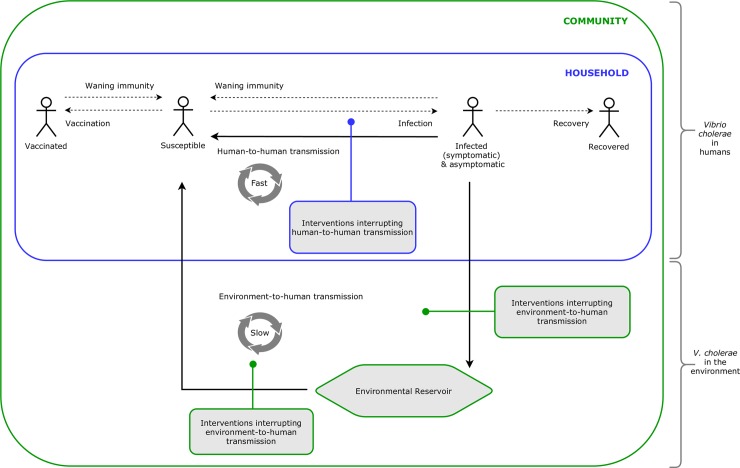
Conceptual framework of cholera transmission within the household and at the community-level: incorporating the human-to-human and environment-to-human pathways of transmission (adapted from recent models [27, 43, 45, 61]).

Moving through the guidelines chronologically, the evidence synthesis consisted of four stages:

- extracting all recommendations from the different guidelines and classifying them according to 11 categories of WASH interventions, consistent with definitions used in previous systematic reviews of WASH interventions [95, 96], listed in Table 1;
- measuring concordance among guidelines, whereby all recommendations within each WASH intervention category were analysed through a Fleiss’ Kappa Statistic (κ) for interrater agreement on a scale from <0 to 1 for perfect agreement [97, 98];
- identifying consistent recommendations, whereby each recommendation was classified as “Recommended” when it featured in the guideline, “Not Recommended” if the guideline clearly stated that the intervention was not recommended by the authors/agency or “Recommendation not listed” if otherwise, see examples in Table 2;
- categorising recommendations on the basis of whether they would interrupt within-household or community-level transmission. The conceptual framework also incorporates key transmission pathways within the two domains of transmission, based on recent models describing human-to-human and environment-to-human transmission of cholera [27, 43, 61, 99], described in Fig 1.

The details of each recommendation, including mode and frequency of intervention delivery, duration of the intervention and any other factors deemed relevant were also noted. A quality assessment for risk of bias among guidelines was not performed. A narrative summary of data extraction and analysis was developed by one investigator (LDG) and then reviewed by all authors.

**Table 1:** Categories and definitions of water, sanitation and hygiene (WASH) interventions included in the review.

WASH intervention category	Definition
**Improving the access to water sources and/or quantity of water**	Any intervention to provide a new and/or improved water supply or distribution system, or both, i.e. to reduce direct and indirect exposure with contaminated water (e.g. installation of piped water supply, hand pumps, boreholes; installation or extension of distribution networks; water trucking or tankers; and, protection of water sources)
**Improving the quality of water: water treatment at source**	Any intervention to improve the microbiological quality of drinking water at the source, including: - assessment and monitoring of water quality i.e. microbiological, chemical and physical quality - removing or inactivating microbiological pathogens (e.g. water source level water treatment systems, filtration, sedimentation, chemical treatment, heat treatment, ultraviolet (UV) radiation or flocculation)
**Improving the quality of water: point of use (POU) and safe storage**	Any intervention to expand use of or improve the microbiological quality of drinking water at the point of use (POU), including: - assessment and monitoring of water quality i.e. microbiological, chemical and physical quality - protecting the microbiological quality of water prior to consumption (e.g. chemical treatment, filtration, heat treatment, flocculation, UV radiation, residual disinfection, protected distribution, improved storage)
**Improving the access to and use of sanitation facilities and reducing exposure to faeces**	Any intervention to introduce, improve or expand the coverage of facilities for the safe management, disposal and treatment of excreta, i.e. to reduce direct and indirect contact with human faeces (e.g. latrine construction, pour flush, composting or water sealed flush toilet, piped sewer system, septic tank, simple pit latrines, VIP latrine, defecation trenches or use of a potty or scoop for the disposal of child faeces)
**Behaviour change interventions to improve personal, domestic and food hygiene practices**	Any intervention to improve hygiene, including: - promotion of hygiene behaviours, norms or practices surrounding personal, food and hand hygiene - assessment and monitoring of hygiene behaviours, norms or practices, including adaptation of activities - any named method of delivery of hygiene promotion (e.g. interpersonal channels, house-to-house visits, community meetings, mass and social media, targeted areas or information, education and communication (IEC) materials, or other hygiene promotion activities) - any named theory, framework or technique for hygiene promotion (e.g. behaviour change communication (BCC), community engagement, social marketing and demand creation, integrated hardware)
**Distribution of hygiene materials or non-food items (NFIs)**	Any intervention that provides hygiene materials or use of hygiene materials (e.g. soap, hygiene kits, handwashing stands, sinks and other facilities)
**Promotion or distribution of disinfection and cleaning of households and community spaces and/or materials**	Any intervention that provides or distributes disinfection materials (e.g. chlorine spraying, disinfection of clothes, disinfectants, disinfection of bedding or vehicles) or promotes household cleaning (e.g. safe laundry practices, cleaning of floors and furniture)
**Improving dead body management and safe funeral practices**	Any intervention to improve safe funeral practices, funeral gatherings and management of corpses in the community
**Improving the management of wastewater and faecal sludge**	Any intervention to improve management of wastewater and faecal sludge
**Provision of interventions that improve solid waste disposal**	Any intervention to improve solid waste disposal, particularly in public places
**Use of vector control interventions to reduce flies**	Any intervention to improve fly control and/or other vectors
**Other WASH interventions**	*As applicable*

**Table 2:** Classifying recommendations, definitions and examples.

Recommendation classification	Definition	Examples of the terminology	Example from the guidelines
**"Recommended"**	"Recommended" interventions were those that were listed in the guideline unless there is rationale not to.	"strongly recommended", "should", "offer", “provide”	"At least 20 litres of potable water should be provided per person and per day for drinking and hygiene (personal and domestic)" MSF 2017
**"Not recommended"**	"Not recommended" interventions applied when there was a strong statement in the guideline of no benefit and/or harms outweighing benefits.	"do not recommend", "do not provide", "not appropriate", "should not", “will not”	“Oxfam GB will not implement, advocate for or support the following as an appropriate response to cholera control: spraying to reduce the number of flies” Oxfam 2012
**"Recommendation not listed"**	"Recommendation not listed" applied when there was no recommendation listed for or against a practice.	n/a	n/a

## Results

### Search results and characteristics of included guidelines

**Fig 2:**
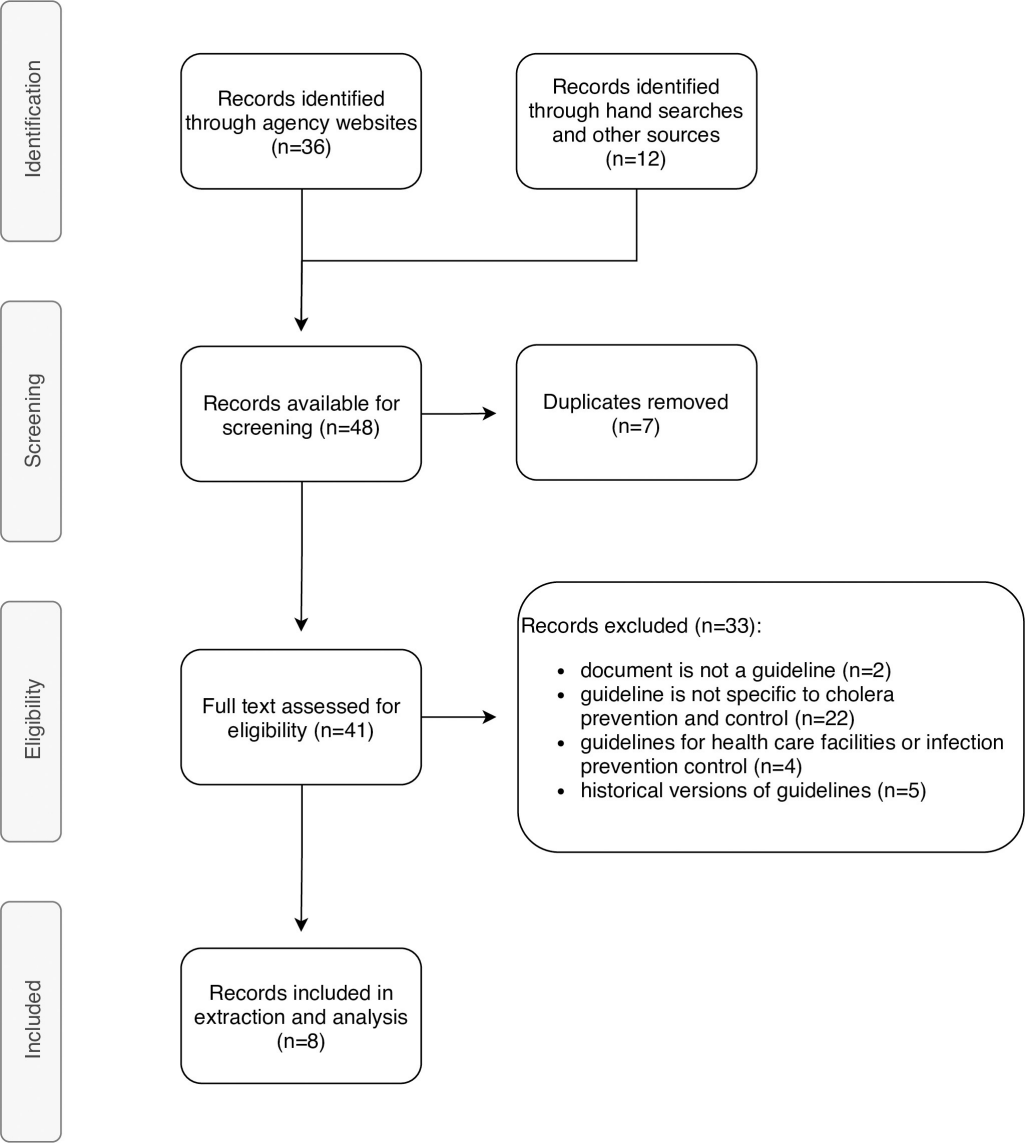
Overview of the search strategy and selection: PRISMA-ScR diagram.

Searches were finalised on 14^th^ February 2019. The search strategy identified a total of 48 records. After de-duplication and screening, eight guidelines met the inclusion criteria for review and are included in this scoping review. The guidelines were published between 2004 to 2019; three were authored by international non-governmental organisations (NGOs)–Médecins Sans Frontières (MSF) [100], Oxfam [101], Action Contre la Faim (ACF) [102]; one from a non-profit organisation (NPO)–the Sphere Project [103]; three by multilateral organisations–United Nation’s Children Fund (UNICEF) [104], the World Health Organization (WHO) [105] and the Global Task Force on Cholera Control (GTFCC) [106]; and one by a research institution–the International Centre for Diarrhoeal Disease Research Bangladesh (ICDDR’B) [107]. The guidelines were published in English (n = 7) and French (n = 1). No guidelines were excluded based on language. All excluded records are listed in S3 Appendix, with reasons for exclusion. The guideline selection process is outlined in Fig 2 and reported according to the PRISMA-ScR checklist [94] in S4 Appendix.

Guidelines were not restricted to specific contexts (epidemic/endemic, urban/rural), except one guideline that was specific for cholera outbreaks in crisis contexts such as conflict settings, natural disasters, refugee camps, and among internally displaced populations or populations on the move [103].

A total of 95 recommendations were extracted. UNICEF (2013) listed the most recommendations (n = 66) [104], followed by ACF (2013) [102], MSF (2017) [100], Sphere (2018) [103] and Oxfam (2012) [101] who all had a similar number of recommendations (n = 54, 53, 53, 51, respectively). Guidelines published by WHO (2004) [105], ICDDR’B (2018) [107] and GTFCC (2019) [106] had the fewest recommendations (n = 26, 34 and 42, respectively).

### Classifying recommendations by WASH intervention categories

Recommendations were classified across 11 categories of WASH interventions (Table 3). Among the 95 recommendations, 32 (34% combined) focused on improving the quantity, access and quality of water, at both source and point of use (POU) and 13 (14%) on improving sanitation access and use. Interventions to improve personal, domestic and food hygiene, such as behaviour change or distribution of non-food items (NFIs), also featured heavily (n = 18 and n = 8, 27% combined). Other, more specific interventions, such as disinfection of households and community spaces or dead body management, featured less frequently (n = 10 and n = 7, or 11% and 7% respectively). Interventions such as management of wastewater and faecal sludge, solid waste disposal and fly control, were infrequently mentioned (n = 3, n = 3 and n = 1, 7% combined).

**Table 3:** Number of recommendations listed by each guideline, classified by WASH intervention category and analysed for concordance among guidelines.

Categories of water, sanitation and hygiene (WASH) interventions	Total (n)	WHO, 2004	Oxfam, 2012	ACF, 2013	UNICEF, 2013	MSF, 2017	Sphere, 2018	ICDDR’B, 2018	GTFCC, 2019	Fleiss Kappa Statistic (κ) for interrater agreement among guidelines		Key to Fig 3.
**Improving the access to water sources and/or quantity of water**	9	3	4	6	6	6	7	4	2	0.19	Slight	1–9
**Improving the quality of water: water treatment at source**	12	3	5	9	4/1NR	7/1NR	6	5	4	0.30	Fair	10–21
**Improving the quality of water: point of use (POU) and safe storage**	11	3	6	9	6	7	8	6	7	0.36	Fair	22–32
**Improving the access to and use of sanitation facilities and reducing exposure to faeces**	13	4	4	3	10	6	10	3	5	0.09	Slight	33–45
**Behaviour change interventions to improve personal, domestic and food hygiene practices**	18	8	13	12	17	8	11	8	12	0.23	Fair	46–63
**Distribution of hygiene materials or non-food items (NFIs)**	8	0	6	4	6	4	5	2	2	0.25	Fair	64–71
**Promotion or distribution of disinfection and cleaning of households and community spaces and/or distribution of materials**	7	1	3NR	2/2NR	4/2NR	4/2NR	1	1	1	0.24	Fair	72–78
**Improving dead body management and safe funeral practices**	10	4	5/1NR	6	7	8	0	5	8	0.08	Slight	79–88
**Improving the management of wastewater and faecal sludge**	3	0	2	1	1	0	2	0	1	0.01	Slight	89–91
**Provision of interventions that improve solid waste disposal**	3	0	1	0	2	0	3	0	0	-0.07	Poor	92–94
**Use of vector control interventions to reduce flies**	1	0	1NR	0	0	0	0	0	0	-0.14	Poor	95
**Total number of recommendations listed in each guideline (n)**	**95**	**26**	**51**	**54**	**66**	**53**	**53**	**34**	**42**	**0.25**	**Fair**	**-**

**Fig 3:**
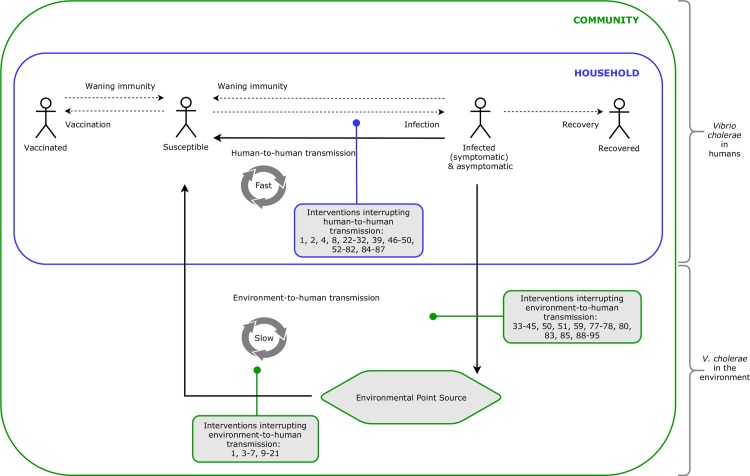
95 recommended WASH interventions found across eight current international guidelines mapped to the conceptual framework of cholera transmission within the household and at the community-level.

NR- Not Recommended by a guideline; “Key to Fig 3.” provides the numbered recommendations to be used with Fig 3; WHO- World Health Organization, MSF- Médecins Sans Frontières, ICDDR’B- International Centre for Diarrhoeal Disease Research Bangladesh, ACF- Action Contre la Faim, UNICEF- United Nations Children’s Fund, GTFCC- Global Task Force on Cholera Control

### Measuring concordance among guidelines

The interrater agreement among guidelines, as to which WASH interventions they proposed, ranged from -0.14 to 0.36 (Fleiss’ Kappa Statistic (κ)), indicating a poor to fair level of agreement among guidelines (Table 3). The mean interrater agreement was slight at 0.14 and overall concordance among guidelines was fair at 0.25.

### Identifying consistently recommended WASH interventions

Twenty consistent recommendations (defined as those mentioned by at least seven of the eight guidelines) were identified (Table 4). These interventions fell under seven of the 11 categories of WASH, and included: improving the access to water sources and/or quantity of water (n = 2); improving the quality of water at source (n = 3); improving the quality of water at point of use (POU) and safe storage (n = 5); behaviour change interventions to improve personal, domestic and food hygiene practices (n = 6); distribution of hygiene materials and non-food items (NFIs) (n = 2); promotion of disinfection or cleaning of households, community spaces and/or distribution of materials (n = 1); and, improving dead body management and safe funeral practices (n = 1). The majority of the consistently recommended interventions (n = 10, 50%) targeted within-household transmission, three targeted community-level transmission (35%) and another seven recommendations targeted both (15%). Additionally, all guidelines recommended that interventions and messages should be adapted to the local context and cultural practices of the population.

**Table 4:** Twenty consistently recommended WASH interventions for cholera prevention and control.

Recommendation	Total (n)	WHO, 2004	Oxfam, 2012	ACF, 2013	UNICEF, 2013	MSF, 2017	Sphere, 2018	ICDDR’B, 2018	GTFCC, 2019	Transmission domain
**Improving the access to water sources and/or quantity of water**										
Assessment and mapping of existing water sources (i.e. availability, types, access, quantity of water, risks of contamination)	8	✓	✓	✓	✓	✓	✓	✓	✓	Household/ Community
Installation or repair of temporary or permanent improved water sources (e.g. boreholes, protected wells, protected hand pumps, protected springs, water tankers, water distribution systems including taps to households or public spaces and/or protection of the water source)	7	✓	✓	✓	✓	✓	✓	×	✓	Household/ Community
**Improving the quality of water: water treatment at source**										
A free residual chlorine (FRC) concentration of >0.5mg/l measured at source	8	✓	✓	✓	✓	✓	✓	✓	✓	Community
Highly turbid water, at source, should not be chlorinated and filtration, coagulation-flocculation or other pre-treatments should be used to reduce turbidity before treatment	7	✓	✓	✓	×	✓	✓	✓	✓	Community
Bulk or batch chlorination of water sources (e.g. in-line chlorination of water distribution systems, temporary bladders, water tanks and trucking), with dosage determined by jar tests	7	✓	✓	✓	✓	✓	✓	✓	×	Community
**Improving the quality of water: point of use (POU) and safe storage**										
Promotion of household water treatment products/technologies	8	✓	✓	✓	✓	✓	✓	✓	✓	Household
Distribution of household water treatment products/technologies	7	×	✓	✓	✓	✓	✓	✓	✓	Household
Promotion of cleaning, coverage and/disinfection of safe water storage containers	7	✓	✓	✓	✓	✓	✓	×	✓	Household
Highly turbid water, at point of use, should not be chlorinated and filtration, coagulation-flocculation or other pre-treatments should be used to reduce turbidity before treatment	7	✓	✓	✓	×	✓	✓	✓	✓	Household
Monitoring of water quality at the household	7	×	✓	✓	✓	✓	✓	✓	✓	Household
**Behaviour change interventions to improve personal, domestic and food hygiene practices**										
Promotion of handwashing after defecation, before eating, before preparing food, before feeding a child, after cleaning a child's faeces and after contact with a cholera case	8	✓	✓	✓	✓	✓	✓	✓	✓	Household
Promotion of safe water collection, treatment and storage (e.g. for drinking and cooking)	7	✓	✓	✓	✓	✓	✓	×	✓	Household
Promotion of safe food preparation, cooking and storage (e.g. covering food to avoid flies and contamination, promotion of breastfeeding)	7	✓	✓	×	✓	✓	✓	✓	✓	Household
Promotion of safe defecation practices (e.g. no open defecation, use of latrines, cleaning of latrines, safe disposal of child faeces)	7	✓	✓	×	✓	✓	✓	✓	✓	Household/ Community
Hygiene promotion through house-to-house visits or community meetings	7	×	✓	✓	✓	✓	✓	✓	✓	Household/ Community
Hygiene promotion and cholera awareness using mass media (e.g. radio, television, SMS, social media)	8	✓	✓	✓	✓	✓	✓	✓	✓	Household/ Community
**Distribution of hygiene materials or non-food items (NFIs)**										
Distribution of soap to households	7	×	✓	✓	✓	✓	✓	✓	✓	Household
Installation of handwashing points in public places (e.g. markets, schools, public toilets)	7	×	✓	✓	✓	✓	✓	✓	✓	Household/ Community
**Promotion and distribution of disinfection and cleaning of households and community spaces and/or distribution of materials**										
Promotion of safe laundry practices, including disinfection of clothes and bedding of cholera cases with chlorine, boiling for 5 minutes or drying in the sun; alternatively burn or bury with the deceased	7	✓	×	✓	✓	✓	✓	✓	✓	Household
**Improving dead body management and safe funeral practices**										
Disinfection of corpses with chlorine, and fill mouth and anus with cotton wool soaked in chlorine	7	✓	✓	✓	✓	✓	×	✓	✓	Household/ Community

✓ - Present in guideline; × - Not found in guideline; “Household” and “Community” denote the two levels of cholera transmission and where WASH interventions would be implemented and used; WHO- World Health Organization, MSF- Médecins Sans Frontières, ICDDR’B- International Centre for Diarrhoeal Disease Research Bangladesh, ACF- Action Contre la Faim, UNICEF- United Nations Children’s Fund, GTFCC- Global Task Force on Cholera Control

Six interventions were explicitly described as not recommended for cholera prevention and control by four organisations [100–102, 104] and all involved the use of chemical products (Table 5). There was clear disagreement and contradictions between the organisations, some of which were based on the lack of available evidence to support interventions, including the provision of disinfection products, chlorine spraying and use of insecticides to control fly populations.

**Table 5:** WASH interventions not recommended for cholera prevention and control by one or more guidelines.

Recommendation	Total (n)	WHO, 2004	Oxfam, 2012	ACF, 2013	UNICEF, 2013	MSF, 2017	Sphere, 2018	ICDDR’B, 2018	GTFCC, 2019	Transmission domain
**Improving the quality of water: water treatment at source**										
Chlorination of unimproved water sources (e.g. unprotected wells, unlined wells)	2NR	×	×	×	NR	NR	×	×	×	Community
**Promotion and distribution of disinfection and cleaning of households and community spaces and/or distribution of materials**										
**Disinfection of households with chlorine spraying (especially vomit and faeces)**	4NR	×	NR	NR	NR	NR	×	×	×	Household
Disinfection of non-households with chlorine spraying (e.g. in vehicles, marketplaces)	4NR	×	NR	NR	NR	NR	×	×	×	Community
Provision of disinfection materials to households for household cleaning and disinfection (e.g. detergents, 0.5–2% chlorine solution)	1NR	×	NR	✓	✓	✓	×	×	×	Household
**Improving dead body management and safe funeral practices**										
Promotion or provision of hygiene materials to households for safe and hygienic corpse preparation (e.g. detergents, 0.5–2% chlorine solution, body bags)	1NR	✓	NR	✓	✓	✓	×	×	×	Household
**Use of vector control interventions to reduce flies**										
**Reduction of fly populations through insecticide spraying in breeding areas**	1NR	×	NR	×	×	×	×	×	×	Community

✓ - Present in guideline; × - Not found in guideline; NR—Not recommended; “Household” and “Community” denote the two levels of cholera transmission and where WASH interventions would be implemented and used; WHO- World Health Organization, MSF- Médecins Sans Frontières, ICDDR’B- International Centre for Diarrhoeal Disease Research Bangladesh, ACF- Action Contre la Faim, UNICEF- United Nations Children’s Fund, GTFCC- Global Task Force on Cholera Control

### Categorising recommendations to conceptual models of cholera transmission

From the 95 recommendations found across guidelines, 33 (35%) would target within-household transmission, 43 (45%) community-level and 19 (20%) would affect both domains (Table 6). Table 6 also describes how many recommendations each guideline made for within-household or community-level interventions.

**Table 6:** Categorisation of WASH recommendations, by each of the eight included guidelines, according to domains of cholera transmission.

Domain of transmission targeted by WASH interventions	Total (n/%)	WHO, 2004 (n/%)	Oxfam, 2012 (n/%)	ACF, 2013 (n/%)	UNICEF, 2013 (n/%)	MSF, 2017 (n/%)	Sphere, 2018 (n/%)	ICDDR’B, 2018 (n/%)	GTFCC, 2019 (n/%)
Within-household	33 (35)	11 (42)	19 (37)	21 (39)	23 (35)	21 (21)	18 (34)	13 (38)	15 (36)
Community-level	43 (45)	7 (27)	19 (37)	21 (39)	27 (41)	20 (38)	24 (45)	10 (30)	13 (31)
Within-household and community-level	19 (20)	8 (31)	13 (25)	12 (22)	16 (24)	12 (23)	11 (21)	11 (32)	14 (33)
**Total recommendations**	**95**	**26**	**51**	**54**	**66**	**53**	**53**	**34**	**42**

WHO- World Health Organization, MSF- Médecins Sans Frontières, ICDDR’B- International Centre for Diarrhoeal Disease Research Bangladesh, ACF- Action Contre la Faim

UNICEF- United Nations Children’s Fund, GTFCC- Global Task Force on Cholera Control

A full list of the 95 recommendations, concordance among guidelines and whether an intervention was categorised to target within-household or community-level transmission, is provided in the supplementary materials (S1 Table). Each of the 95 recommendations listed in S1 Table has been mapped to the conceptual framework of cholera transmission in Fig 3 (with the numbers in Table 3 acting as a key to the recommendations), including the theoretical interruption of human-to-human or environment-to-human cholera transmission.

## Discussion

Our scoping review of current international guidelines found that guidelines generally recommend all categories of WASH interventions for cholera prevention and control, with 95 distinct recommendations extracted from the eight included guidelines. The guidelines had poor to fair concordance, and some had considerably fewer recommendations than others. Among the 95 recommendations identified, 20 recommendations were consistently recommended by seven or more guidelines. Overall, the guidelines proposed a balance between interventions addressing within-household and community-level transmission of cholera, however, the majority of guidelines focused on community-level interventions. We anticipate that undertaking this scoping review of WASH guidelines for cholera prevention and control has the potential to be a useful tool for both implementing organisations and national governments to further develop and guide response strategies. Particularly as our findings suggest that guidelines, notably those written by multilateral agencies and informing national policy, require more structured alignment, and, in terms of WASH interventions, should consider how interventions effectively reduce transmission pathways, as well as economic and feasibility criteria, when making recommendations for the prevention and control of cholera.

Improvements to personal, domestic and food hygiene, water quantity and quality were the most consistently recommended interventions, many of which targeted within-household transmission of cholera. Accordingly, all, or some subset of, the 20 consistently recommended WASH interventions could be considered as the “minimum standard” interventions that organisations have proposed for effective cholera response programmes. Neither hygiene nor water improvements are new to public health nor to cholera control [1, 70, 108–110], but in addition to controlling cholera outbreaks, these interventions could prevent recurrent epidemics in endemic areas. Additionally, if governments and organisations move away from disease-specific efforts and towards systems strengthening, these interventions may be viewed in terms of their broader effects on WASH-related diseases and other health outcomes [111, 112].

A high number of recommendations does not necessarily render guidelines more useful or more likely to be used. Fewer, more focused recommendations may mitigate the potential for confusion at an operational level and incentivise uptake. To an extent, the low concordance among guidelines observed in this review could indicate the potential difficulty of using the available guidelines, by practitioners and policy makers, to decide which interventions to propose or which guidelines to follow. It may also disincentivise uptake or confuse the prioritisation of interventions among implementers. Only half of the included guidelines explicitly discouraged specific interventions, which in practice may be helpful to concentrate efforts and reduce the range of options considered. On the other hand, interventions that have not been recommended may point to mixed, inconclusive or low-quality evidence. During this review, we did not assess which of the interventions were based on concrete or published evidence. There is a lack of evidence regarding the effectiveness of the interventions across the guidelines, as well as concerns around timeliness, prioritisation of other interventions, cost-effectiveness and potential stigmatisation of the beneficiaries [101, 104]. All of which implies evidence to support the recommendations listed are an area requiring further work.

### Effective interventions to reduce within-household transmission

Considering recent evidence on the heightened risk of intra- and interhousehold transmission of cholera, reactive interventions to control and contain cholera outbreaks should take advantage of this clustering by targeting cases, their households and the associated human-to-human transmission pathway [28, 67]. Most recommendations in the included guidelines did address this pathway (35% targeting within-household and 20% targeting both within-household and community-level transmission), and generally reflected new evidence of effective transmission reduction through household-level interventions [44, 47, 49, 63]. However, effective delivery strategies or modalities for implementation of household-level interventions, such as recently introduced case-centred models for the delivery of interventions (i.e. CATIs) or HCF-based strategies for delivery of interventions, were rarely discussed. Limited attention was given to the importance of responding rapidly [44, 113], particularly due to the hyper infective nature of newly shed *V*. *cholerae* from cholera cases [114] and lower infective dose required for transmission from cases in the first days of bacterial shedding [60], or repeated delivery of interventions [115, 116], which are all important considerations for effective disease reduction.

Behaviour change interventions were among the only recommendations for which the modality of delivery was specified, e.g. “*Hygiene promotion through house-to-house visits or community meetings*” and “*Hygiene promotion and cholera awareness using mass media (e*.*g*. *radio*, *television*, *SMS and social media)*”. Whilst there is some evidence to support radio as a preferred or trusted communication means in cholera outbreaks [110], guidelines would benefit from more explicitly incorporating the evidence base on the other delivery modalities and platforms available. Behaviour change interventions that were recommended across the guidelines should also consider the limited effect of health education and messaging alone [117–119], and incorporate activities to improve the role of collective or community engagement in response activities [111, 120]. Recommendations should rely on the available evidence base to design context-specific behaviour change interventions, including evidence from non-outbreak settings, that facilitate WASH intervention uptake [121], with an emphasis placed on assessing practices in the population before proposing set strategies, and allowing programmes to adapt and change according to needs.

Available evidence also suggests that case-centred strategies or CATIs, which require targeting fewer people per case averted and delivery of interventions centred to cases, are more cost-effective and resource-efficient for delivery of interventions [1, 44, 49, 67, 122, 123]. For example, hygiene and health promotion and the distribution of hygiene kits at the point of care have been observed as an effective delivery channel in cholera control [63, 78, 113], and in other disease reduction efforts [124–126], yet recommendations on the location of intervention delivery was either omitted or limited in all eight of the guidelines. Prepositioning of supplies for distribution has also been noted as an important consideration to allow for timely response in case-centred and mass-delivered strategies [110].

### Effective interventions to reduce community-level transmission

Cholera affects communities already burdened with a lack of infrastructure, poor health systems and affected by crises. Any global map of poor water and sanitation services, and high levels of poverty and insecurity, is essentially the same as the map of cholera burden [1, 21]. Although models have highlighted within-household and human-to-human transmission as the catalyst in epidemics, interventions that target community-level transmission and the environment-to-human transmission pathways remain important for cholera prevention. Regional resurgences of cholera are a contributing factor to the burden of disease globally [21, 23, 24, 127], with notable high incidence of disease and recurrent outbreaks in the lacustrine areas of East and Central Africa [128–130]. Community-level or mass population strategies in areas such as this may limit the reliance on active case finding or attendance at HCFs required by case-centred approaches, and provide interventions that also target the estimated 40 to 80% of cholera cases which are asymptomatic [19, 131]. Ultimately, the elimination of cholera can only happen by limiting exposure to or reinfection from a contaminated environment for the entire population [1, 64, 108, 132].

Historically, improvements to WASH infrastructure at a population level such as the community-level interventions listed across the included guidelines, have reduced the incidence of cholera, and other diarrhoeal diseases [111, 120, 133–137], and eliminated the disease since the time of John Snow [108, 109, 138]. However, guidelines reviewed offered little specificity on the standards that should be attained for these WASH interventions. For example, water quality at source is reliant on meeting minimum quality standards such as “*A free residual chlorine concentration of >0*.*5 mg/l measured at source*” and “*A turbidity less than 5 NTU at the water source*, *up to 20 NTU acceptable*” [139]. However, guidelines did not consistently state specific corresponding standards for other WASH interventions such as water availability. Given evidence that limited hours of water availability during the day [140], distance and time needed to fetch water [111, 141] all affect health and water-use practices negatively, standards for water availability, and other WASH interventions, should be further specified across their included recommendations. By contrast, levels and standards of WASH service provision (e.g. ‘limited’, ‘basic’, ‘safely managed’) are more explicitly stated in the SDG indicators and targets [142–146]. The current recommendations in the guidelines to reduce community-level transmission may be more aligned to the first phases of an outbreak whereas the SDG-type standards for these interventions would be required for the longer-term strategy for prevention of outbreaks. Regardless, all recommendations for both community-level and within-household interventions for the prevention or control of cholera require further alignment to national and international targets for WASH service delivery.

### Limitations

Our review only included current, international and accessible guidelines for the prevention and control of cholera. This may have affected how many recommendations were found and the review will have excluded any context specific or more detailed interventions from national guidelines and other sources. The review also does not systematically address the level of evidence supporting the different recommendations, and does not factor in which interventions would be more effective at reducing transmission than others.

In this review, we have considered the risk of transmission within two domains: within-household and at the community-level. Although the separation of household and community is potentially more intuitive for practitioners and policymakers to understand and use, the conceptual cholera transmission framework may diminish the observed overlap of household and community-level transmission and the associated human-to-human and environment-to-human transmission pathways. Neither domains nor pathways of transmission are dichotomous and, aside from interpersonal contact, there is a close association of the risk factors among levels. Human-to-human transmission, or interpersonal contact between infected and susceptible cases, will also occur outside of the household (e.g. in mass gatherings, community places) [28]. Additionally, regular cholera outbreaks in endemic settings may be associated with seasonal climatic patterns (e.g. temperature and humidity) [147, 148], and epidemic cholera is often triggered by weather conditions [149], such that it changes households’ behaviours (e.g. water collection practices) and interaction with the aquatic environment which in turn increases the risk of community-level environment-to-human transmission [25].

Concordance or consistency of recommendations is not necessarily a measure of guideline quality, but rather of how much agreement there is among guidelines. Concordance scores may simply reflect a lack of detail or prioritisation of certain service areas, rather than explicit decisions to include specific interventions. Nevertheless, the less agreement, the more potential there is for inappropriate interventions or conflicted decision-making among national governments and responding organisations, and the more likely it is that evidence has not been considered systematically when developing guidelines [93, 150], suggesting a need for greater scientific and policy collaboration among organisations.

None of the guidelines explicitly stated their process for guideline development such as using the GRADE system [151, 152] or other recommended methods [93, 150, 153, 154] to determine the quality of evidence for each recommendation. Any new development of guidelines should either use and adhere to these recommended processes to strengthen their quality and use, or clearly describe their methods. Additionally, as the objectives of this review did not include an assessment of guideline quality, readers may come away not understanding guideline quality or which, if any, of these guidelines should be considered in cholera programmes. However, the review was not intended to make this decision as we are unable to take into account the specific mission or mandate of each author organisation, which may affect the priority given to different types of intervention or indeed WASH as a whole.

### Conclusions and recommendations

The Global Roadmap for Cholera Elimination by 2030 has focused attention on current efforts to prevent and control cholera [68], and highlighted the need for clear, consistent and evidence-based guidelines. A number of international guidelines for cholera prevention and control are in current use; however, the concordance among the WASH recommendations in these guidelines was relatively low. Overall, the guidelines did propose a balance of interventions to reduce within-household and community-level transmission. Interventions to reduce within-household transmission were consistently proposed and could be a minimum package of interventions to address outbreak control. Interventions to reduce community-level transmission tended to interrupt transmission between a contaminated environment and susceptible individuals or contamination of the environment by cases, but did not often specify the level of service that should be provided to reduce transmission. Guidelines should more explicitly consider strength of evidence, efficiency and feasibility criteria when recommending different candidate WASH interventions.

No single guideline included all recommendations or collated all available guidance. Interpretation of the guidelines may be difficult particularly where recommendations are omitted or contradict one another. Based on this review, we make five recommendations to strengthen the development of future guidelines for cholera prevention and control:

- Considering the different phases of cholera outbreaks, WASH interventions should target human-to-human transmission within the household and at the community-level for outbreak control, and environment-to-human transmission at a community-level for cholera prevention in recurrent settings and areas where reinfection during outbreaks is likely;
- Limiting the number of guidelines available and compiling fewer, more focused recommendations in guidelines so as to mitigate the potential for confusion at an operational level and incentivise uptake;
- Providing greater specificity in the language used in recommendations, e.g. specifying the timing of response, coverage required, minimum levels of service and modality of delivery (e.g. location, population group);
- Publishing or improving access to programme evaluations and practice literature to strengthen the evidence base for guideline development, and to support national cholera control plans as part of the Global Roadmap for Cholera Elimination by 2030;
- Standardising approaches in guideline development to consider the evidence base, from studies, programme evaluations or models, when deciding which interventions to recommend.

## Supporting information

S1 Table All recommendations found across guidelines. (DOCX)

S1 Appendix Search strategy and resources searched. (DOCX)

S2 Appendix Search terms. (DOCX)

S3 Appendix Excluded guidelines. (DOCX)

S4 Appendix PRISMA-ScR checklist. (DOCX)

## References

[1] Legros D, Partners of the Global Task Force on Cholera C. Global Cholera Epidemiology: Opportunities to Reduce the Burden of Cholera by 2030. J Infect Dis. 2018;218(suppl_3):S137–S40. Epub 2018/09/06. 10.1093/infdis/jiy486 30184102 PMC6207143

[2] Spiegel PB, Le P, Ververs MT, Salama P. Occurrence and overlap of natural disasters, complex emergencies and epidemics during the past decade (1995–2004). Confl Health. 2007;1:2 10.1186/1752-1505-1-2 17411460 PMC1847810

[3] Checchi F, Gayer M, Freeman Grais R, Mills EJ. Public health in crises-affected populations: a practical guide for decision-makers. London, UK: Humanitarian Practice Network at ODI, 2007.

[4] Shannon K, Hast M, Azman AS, Legros D, McKay H, Lessler J. Cholera prevention and control in refugee settings: Successes and continued challenges. PLoS Negl Trop Dis. 2019;13(6):e0007347 Epub 2019/06/21. 10.1371/journal.pntd.0007347 31220084 PMC6586254

[5] Connolly MA, Gayer M, Ryan MJ, Salama P, Spiegel P, Heymann DL. Communicable diseases in complex emergencies: impact and challenges. Lancet. 2004;364(9449):1974–83. 10.1016/S0140-6736(04)17481-3 . 15567014

[6] Connolly MA, Heymann DL. Deadly comrades: war and infectious diseases. Lancet. 2002;360 Suppl:s23–4. 10.1016/s0140-6736(02)11807-1 . 12504490

[7] Cronin AA, Shrestha D, Cornier N, Abdalla F, Ezard N, Aramburu C. A review of water and sanitation provision in refugee camps in association with selected health and nutrition indicators–the need for integrated service provision. Journal of Water and Health. 2008;6(1):1–13. 10.2166/wh.2007.019 17998603

[8] Cronin AA, Shrestha D, Spiegel P, Gore F, Hering H. Quantifying the burden of disease associated with inadequate provision of water and sanitation in selected sub-Saharan refugee camps. Journal of Water and Health. 2009;7(4):557–68. 10.2166/wh.2009.089 19590123

[9] Gayer M, Legros D, Formenty P, Connolly MA. Conflict and emerging infectious diseases. Emerg Infect Dis. 2007;13(11):1625–31. 10.3201/eid1311.061093 18217543 PMC3375795

[10] Sack DA, Sack RB, Nair GB, Siddique AK. Cholera. Lancet. 2004;363(9404):223–33. 10.1016/s0140-6736(03)15328-7 . 14738797

[11] Shikanga OT, Mutonga D, Abade M, Amwayi S, Ope M, Limo H, et al High mortality in a cholera outbreak in western Kenya after post-election violence in 2008. Am J Trop Med Hyg. 2009;81(6):1085–90. Epub 2009/12/10. 10.4269/ajtmh.2009.09-0400 . 19996441

[12] Spiegel P, Sheik M, Gotway-Crawford C, Salama P. Health programmes and policies associated with decreased mortality in displaced people in postemergency phase camps: a retrospective study. Lancet. 2002;360(9349):1927–34. 10.1016/S0140-6736(02)11915-5 . 12493259

[13] Spiegel PB, Checchi F, Colombo S, Paik E. Health-care needs of people affected by conflict: future trends and changing frameworks. Lancet. 2010;375(9711):341–5. 10.1016/S0140-6736(09)61873-0 . 20109961

[14] Toole MJ, Waldman RJ. Prevention of excess mortality in refugee and displaced populations in developing countries. JAMA. 1990;263(24):3296–302. . 2348541

[15] Smith KF, Goldberg M, Rosenthal S, Carlson L, Chen J, Chen C, et al Global rise in human infectious disease outbreaks. J R Soc Interface. 2014;11(101):20140950 10.1098/rsif.2014.0950 25401184 PMC4223919

[16] WHO. Disease outbreaks archive 1996 to present [02/03/17]. Available from: http://www.who.int/csr/don/archive/year/en/.

[17] Ganesan D, Gupta SS, Legros D. Cholera surveillance and estimation of burden of cholera. Vaccine. 2019 Epub 2019/07/22. 10.1016/j.vaccine.2019.07.036 . 31326254

[18] WHO. Cholera 2017. Weekly Epidemiological Record. 2018.

[19] Harris JB, LaRocque RC, Qadri F, Ryan ET, Calderwood SB. Cholera. Lancet. 2012;379(9835):2466–76. 10.1016/S0140-6736(12)60436-X 22748592 PMC3761070

[20] Ali M, Nelson AR, Lopez AL, Sack DA. Updated global burden of cholera in endemic countries. PLoS Negl Trop Dis. 2015;9(6):e0003832 Epub 2015/06/05. 10.1371/journal.pntd.0003832 26043000 PMC4455997

[21] Lessler J, Moore SM, Luquero FJ, McKay HS, Grais R, Henkens M, et al Mapping the burden of cholera in sub-Saharan Africa and implications for control: an analysis of data across geographical scales. Lancet. 2018 Epub 2018/03/06. 10.1016/S0140-6736(17)33050-7 . 29502905 PMC5946088

[22] Blake A, Keita VS, Sauvageot D, Saliou M, Njanpop BM, Sory F, et al Temporo-spatial dynamics and behavioural patterns of 2012 cholera epidemic in the African mega-city of Conakry, Guinea. Infect Dis Poverty. 2018;7(1):13 Epub 2018/02/17. 10.1186/s40249-018-0393-8 29448965 PMC5815196

[23] Moore S, Dongdem AZ, Opare D, Cottavoz P, Fookes M, Sadji AY, et al Dynamics of cholera epidemics from Benin to Mauritania. PLoS Negl Trop Dis. 2018;12(4):e0006379 Epub 2018/04/10. 10.1371/journal.pntd.0006379 . 29630632 PMC5908202

[24] Weill FX, Domman D, Njamkepo E, Tarr C, Rauzier J, Fawal N, et al Genomic history of the seventh pandemic of cholera in Africa. Science. 2017;358(6364):785–9. Epub 2017/11/11. 10.1126/science.aad5901 . 29123067

[25] Islam MS, Zaman MH, Islam MS, Ahmed N, Clemens JD. Environmental reservoirs of Vibrio cholerae. Vaccine. 2019 Epub 2019/07/10. 10.1016/j.vaccine.2019.06.033 . 31285087

[26] Tien JH, Earn DJ. Multiple transmission pathways and disease dynamics in a waterborne pathogen model. Bull Math Biol. 2010;72(6):1506–33. 10.1007/s11538-010-9507-6 . 20143271

[27] Fung IC. Cholera transmission dynamic models for public health practitioners. Emerg Themes Epidemiol. 2014;11(1):1 10.1186/1742-7622-11-1 24520853 PMC3926264

[28] Richterman A, Sainvilien DR, Eberly L, Ivers LC. Individual and Household Risk Factors for Symptomatic Cholera Infection: A Systematic Review and Meta-analysis. J Infect Dis. 2018;218(suppl_3):S154–S64. Epub 2018/08/24. 10.1093/infdis/jiy444 30137536 PMC6188541

[29] Deen J, Mengel MA, Clemens JD. Epidemiology of cholera. Vaccine. 2019 Epub 2019/08/10. 10.1016/j.vaccine.2019.07.078 . 31395455

[30] Shapiro RL, Otieno MR, Adcock PM, Phillips-Howard PA, Hawley WA, Kumar L, et al Transmission of epidemic Vibrio cholerae O1 in rural western Kenya associated with drinking water from Lake Victoria: an environmental reservoir for cholera? Am J Trop Med Hyg. 1999;60(2):271–6. 10.4269/ajtmh.1999.60.271 . 10072150

[31] Swerdlow DL, Malenga G, Begkoyian G, Nyangulu D, Toole M, Waldman RJ, et al Epidemic cholera among refugees in Malawi, Africa: treatment and transmission. Epidemiol Infect. 1997;118(3):207–14. Epub 1997/06/01. 10.1017/s0950268896007352 9207730 PMC2808810

[32] Nguyen VD, Sreenivasan N, Lam E, Ayers T, Kargbo D, Dafae F, et al Cholera epidemic associated with consumption of unsafe drinking water and street-vended water—Eastern Freetown, Sierra Leone, 2012. Am J Trop Med Hyg. 2014;90(3):518–23. Epub 2014/01/29. 10.4269/ajtmh.13-0567 24470563 PMC3945698

[33] Acosta CJ, Galindo CM, Kimario J, Senkoro K, Urassa H, Casals C, et al Cholera outbreak in southern Tanzania: risk factors and patterns of transmission. Emerg Infect Dis. 2001;7(3 Suppl):583–7. 10.3201/eid0707.010741 11485679 PMC2631835

[34] DuBois AE, Sinkala M, Kalluri P, Makasa-Chikoya M, Quick RE. Epidemic cholera in urban Zambia: hand soap and dried fish as protective factors. Epidemiol Infect. 2006;134(6):1226–30. 10.1017/S0950268806006273 16623992 PMC2870514

[35] Moradi G, Rasouli MA, Mohammadi P, Elahi E, Barati H. A cholera outbreak in Alborz Province, Iran: a matched case-control study. Epidemiol Health. 2016;38:e2016018 10.4178/epih.e2016018 27188308 PMC4967910

[36] Ujjiga TT, Wamala JF, Mogga JJ, Othwonh TO, Mutonga D, Kone-Coulibaly A, et al Risk Factors for Sustained Cholera Transmission, Juba County, South Sudan, 2014. Emerg Infect Dis. 2015;21(10):1849–52. Epub 2015/09/25. 10.3201/eid2110.142051 26402715 PMC4593433

[37] Burrowes V, Perin J, Monira S, Sack D, Rashid MU, Mahamud T, et al Risk Factors for Household Transmission of Vibrio cholerae in Dhaka, Bangladesh (CHoBI7 Trial). Am J Trop Med Hyg. 2017. doi: 10.4269/ajtmh.16-0871. PMC5462576 28719281

[38] Fredrick T, Ponnaiah M, Murhekar MV, Jayaraman Y, David JK, Vadivoo S, et al Cholera outbreak linked with lack of safe water supply following a tropical cyclone in Pondicherry, India, 2012. J Health Popul Nutr. 2015;33(1):31–8. Epub 2015/05/23. 25995719 PMC4438646

[39] Grandesso F, Allan M, Jean-Simon PS, Boncy J, Blake A, Pierre R, et al Risk factors for cholera transmission in Haiti during inter-peak periods: insights to improve current control strategies from two case-control studies. Epidemiol Infect. 2014;142(8):1625–35. 10.1017/S0950268813002562 . 24112364 PMC9151226

[40] Rodrigues A, Sandstrom A, Ca T, Steinsland H, Jensen H, Aaby P. Protection from cholera by adding lime juice to food—results from community and laboratory studies in Guinea-Bissau, West Africa. Trop Med Int Health. 2000;5(6):418–22. 10.1046/j.1365-3156.2000.00575.x . 10929141

[41] Rashid MU, Rahman Z, Burrowes V, Perin J, Mustafiz M, Monira S, et al Rapid dipstick detection of Vibrio cholerae in household stored and municipal water in Dhaka, Bangladesh: CHoBI7 trial. Trop Med Int Health. 2017;22(2):205–9. 10.1111/tmi.12797 . 27754582

[42] Rashid MU, George CM, Monira S, Mahmud T, Rahman Z, Mustafiz M, et al Chlorination of Household Drinking Water Among Cholera Patients' Households to Prevent Transmission of Toxigenic Vibrio cholerae in Dhaka, Bangladesh: CHoBI7 Trial. Am J Trop Med Hyg. 2016;95(6):1299–304. 10.4269/ajtmh.16-0420 27698273 PMC5154443

[43] Sugimoto JD, Koepke AA, Kenah EE, Halloran ME, Chowdhury F, Khan AI, et al Household Transmission of Vibrio cholerae in Bangladesh. PLOS Neglected Tropical Diseases. 2014;8(11):e3314 10.1371/journal.pntd.0003314 25411971 PMC4238997

[44] Rebaudet S, Bulit G, Gaudart J, Michel E, Gazin P, Evers C, et al The case-area targeted rapid response strategy to control cholera in Haiti: a four-year implementation study. PLoS Negl Trop Dis. 2019;13(4):e0007263 Epub 2019/04/17. 10.1371/journal.pntd.0007263 . 30990822 PMC6485755

[45] Mukandavire Z, Morris JG. Modeling the Epidemiology of Cholera to Prevent Disease Transmission in Developing Countries. Microbiology spectrum. 2015;3(3):10.1128/microbiolspec.VE-0011-2014. 10.1128/microbiolspec.VE-0011-2014 PMC4634708. 26185087 PMC4634708

[46] Wagner EG, Lanoix JN. Excreta disposal for rural areas and small communities. Monogr Ser World Health Organ. 1958;39:1–182. . 13581743

[47] Codeço CT, Coelho FC. Trends in cholera epidemiology. PLoS Med. 2006;3(1):e42 10.1371/journal.pmed.0030042 16435891 PMC1360632

[48] Debes AK, Ali M, Azman AS, Yunus M, Sack DA. Cholera cases cluster in time and space in Matlab, Bangladesh: implications for targeted preventive interventions. Int J Epidemiol. 2016 10.1093/ije/dyw267 . 27789673

[49] Finger F, Bertuzzo E, Luquero FJ, Naibei N, Touré B, Allan M, et al The potential impact of case-area targeted interventions in response to cholera outbreaks: A modeling study. PLOS Medicine. 2018;15(2):e1002509 10.1371/journal.pmed.1002509 29485987 PMC5828347

[50] Bi Q, Azman AS, Satter SM, Khan AI, Ahmed D, Riaj AA, et al Micro-scale Spatial Clustering of Cholera Risk Factors in Urban Bangladesh. PLoS Negl Trop Dis. 2016;10(2):e0004400 Epub 2016/02/13. 10.1371/journal.pntd.0004400 26866926 PMC4750854

[51] Chowdhury F, Mather AE, Begum YA, Asaduzzaman M, Baby N, Sharmin S, et al Vibrio cholerae Serogroup O139: Isolation from Cholera Patients and Asymptomatic Household Family Members in Bangladesh between 2013 and 2014. PLoS Negl Trop Dis. 2015;9(11):e0004183 Epub 2015/11/13. 10.1371/journal.pntd.0004183 26562418 PMC4642977

[52] Weil AA, Begum Y, Chowdhury F, Khan AI, Leung DT, LaRocque RC, et al Bacterial shedding in household contacts of cholera patients in Dhaka, Bangladesh. Am J Trop Med Hyg. 2014;91(4):738–42. 10.4269/ajtmh.14-0095 25114012 PMC4183396

[53] Phelps MD, Azman AS, Lewnard JA, Antillon M, Simonsen L, Andreasen V, et al The importance of thinking beyond the water-supply in cholera epidemics: A historical urban case-study. PLoS Negl Trop Dis. 2017;11(11):e0006103 Epub 2017/11/28. 10.1371/journal.pntd.0006103 29176791 PMC5720805

[54] Blackburn JK, Diamond U, Kracalik IT, Widmer J, Brown W, Morrissey BD, et al Household-level spatiotemporal patterns of incidence of cholera, Haiti, 2011. Emerg Infect Dis. 2014;20(9):1516–9. Epub 2014/08/26. 10.3201/eid2009.131882 25148590 PMC4178390

[55] Domman D, Chowdhury F, Khan AI, Dorman MJ, Mutreja A, Uddin MI, et al Defining endemic cholera at three levels of spatiotemporal resolution within Bangladesh. Nat Genet. 2018;50(7):951–5. Epub 2018/06/27. 10.1038/s41588-018-0150-8 29942084 PMC6283067

[56] George CM, Hasan K, Monira S, Rahman Z, Saif-Ur-Rahman KM, Rashid MU, et al A prospective cohort study comparing household contact and water Vibrio cholerae isolates in households of cholera patients in rural Bangladesh. PLoS Negl Trop Dis. 2018;12(7):e0006641 Epub 2018/07/28. 10.1371/journal.pntd.0006641 30052631 PMC6063393

[57] Rafique R, Rashid MU, Monira S, Rahman Z, Mahmud MT, Mustafiz M, et al Transmission of Infectious Vibrio cholerae through Drinking Water among the Household Contacts of Cholera Patients (CHoBI7 Trial). Front Microbiol. 2016;7:1635 10.3389/fmicb.2016.01635 27803695 PMC5067524

[58] George CM, Rashid M, Almeida M, Saif-Ur-Rahman KM, Monira S, Bhuyian MSI, et al Genetic relatedness of Vibrio cholerae isolates within and between households during outbreaks in Dhaka, Bangladesh. BMC Genomics. 2017;18(1):903 Epub 2017/11/28. 10.1186/s12864-017-4254-9 29178823 PMC5702050

[59] Andrews JR, Basu S. Transmission dynamics and control of cholera in Haiti: an epidemic model. Lancet. 2011;377(9773):1248–55. 10.1016/S0140-6736(11)60273-0 21414658 PMC3172163

[60] Hartley DM, Morris JG Jr., Smith DL. Hyperinfectivity: a critical element in the ability of V. cholerae to cause epidemics? PLoS Med. 2006;3(1):e7 10.1371/journal.pmed.0030007 16318414 PMC1298942

[61] Codeço CT. Endemic and epidemic dynamics of cholera: the role of the aquatic reservoir. BMC Infect Dis. 2001;1:1 10.1186/1471-2334-1-1 11208258 PMC29087

[62] Grad YH, Miller JC, Lipsitch M. Cholera modeling: challenges to quantitative analysis and predicting the impact of interventions. Epidemiology. 2012;23(4):523–30. 10.1097/EDE.0b013e3182572581 22659546 PMC3380087

[63] George CM, Monira S, Sack DA, Rashid MU, Saif-Ur-Rahman KM, Mahmud T, et al Randomized Controlled Trial of Hospital-Based Hygiene and Water Treatment Intervention (CHoBI7) to Reduce Cholera. Emerg Infect Dis. 2016;22(2):233–41. Epub 2016/01/27. 10.3201/eid2202.151175 26811968 PMC4734520

[64] Montgomery M, Jones MW, Kabole I, Johnston R, Gordon B. No end to cholera without basic water, sanitation and hygiene. Bull World Health Organ. 2018;96(6):371-A Epub 2018/06/16. 10.2471/BLT.18.213678 29904216 PMC5996206

[65] Azman AS, Parker LA, Rumunu J, Tadesse F, Grandesso F, Deng LL, et al Effectiveness of one dose of oral cholera vaccine in response to an outbreak: a case-cohort study. Lancet Glob Health. 2016;4(11):e856–e63. 10.1016/S2214-109X(16)30211-X . 27765293

[66] Khan MU, Shahidullah M. Role of water and sanitation in the incidence of cholera in refugee camps. Trans R Soc Trop Med Hyg. 1982;76(3):373–7. Epub 1982/01/01. 10.1016/0035-9203(82)90194-8 . 7112660

[67] von Seidlein L, Deen JL. Preventing cholera outbreaks through early targeted interventions. PLoS Med. 2018;15(2):e1002510 Epub 2018/02/28. 10.1371/journal.pmed.1002510 29485984 PMC5828352

[68] Global Task Force on Cholera Control. Ending Cholera: A Global Roadmap to 2030. 2017.

[69] Najnin N, Leder K, Qadri F, Forbes A, Unicomb L, Winch PJ, et al Impact of adding hand-washing and water disinfection promotion to oral cholera vaccination on diarrhoea-associated hospitalization in Dhaka, Bangladesh: evidence from a cluster randomized control trial. Int J Epidemiol. 2017;46(6):2056–66. Epub 2017/10/13. 10.1093/ije/dyx187 29025064 PMC5837384

[70] Taylor DL, Kahawita TM, Cairncross S, Ensink JH. The Impact of Water, Sanitation and Hygiene Interventions to Control Cholera: A Systematic Review. PLoS One. 2015;10(8):e0135676 Epub 2015/08/19. 10.1371/journal.pone.0135676 26284367 PMC4540465

[71] Huq A, Yunus M, Sohel SS, Bhuiya A, Emch M, Luby SP, et al Simple sari cloth filtration of water is sustainable and continues to protect villagers from cholera in Matlab, Bangladesh. MBio. 2010;1(1). Epub 2010/08/07. 10.1128/mBio.00034-10 20689750 PMC2912662

[72] Colwell RR, Huq A, Islam MS, Aziz KM, Yunus M, Khan NH, et al Reduction of cholera in Bangladeshi villages by simple filtration. Proc Natl Acad Sci U S A. 2003;100(3):1051–5. Epub 2003/01/17. 10.1073/pnas.0237386100 12529505 PMC298724

[73] Conroy RM, Meegan ME, Joyce T, McGuigan K, Barnes J. Solar disinfection of drinking water protects against cholera in children under 6 years of age. Arch Dis Child. 2001;85(4):293–5. Epub 2001/09/25. 10.1136/adc.85.4.293 11567937 PMC1718943

[74] Deb BC, Sircar BK, Sengupta PG, De SP, Mondal SK, Gupta DN, et al Studies on interventions to prevent eltor cholera transmission in urban slums. Bull World Health Organ. 1986;64(1):127–31. Epub 1986/01/01. 3488134 PMC2490926

[75] Azurin JC, Alvero M. Field evaluation of environmental sanitation measures against cholera. Bull World Health Organ. 1974;51(1):19–26. Epub 1974/01/01. 4549038 PMC2366240

[76] Lantagne D, Yates T. Household Water Treatment and Cholera Control. J Infect Dis. 2018;218(suppl_3):S147–S53. Epub 2018/09/15. 10.1093/infdis/jiy488 30215739 PMC6188534

[77] Patrick M, Berendes D, Murphy J, Bertrand F, Husain F, Handzel T. Access to safe water in rural Artibonite, Haiti 16 months after the onset of the cholera epidemic. Am J Trop Med Hyg. 2013;89(4):647–53. 10.4269/ajtmh.13-0308 24106191 PMC3795094

[78] Gartley M, Valeh P, de Lange R, Dicarlo S, Viscusi A, Lenglet A, et al Uptake of household disinfection kits as an additional measure in response to a cholera outbreak in urban areas of Haiti. J Water Health. 2013;11(4):623–8. 10.2166/wh.2013.050 . 24334836

[79] Lantagne DS, Clasen TF. Use of Household Water Treatment and Safe Storage Methods in Acute Emergency Response: Case Study Results from Nepal, Indonesia, Kenya, and Haiti. Environmental Science & Technology. 2012;46(20):11352–60. WOS:000309805000065. 22963031 10.1021/es301842u

[80] Cavallaro EC, Harris JR, da Goia MS, dos Santos Barrado JC, da Nobrega AA, de Alvarenga de Junior IC, et al Evaluation of pot-chlorination of wells during a cholera outbreak, Bissau, Guinea-Bissau, 2008. J Water Health. 2011;9(2):394–402. 10.2166/wh.2011.122 . 21942203

[81] Beau De Rochars VE, Tipret J, Patrick M, Jacobson L, Barbour KE, Berendes D, et al Knowledge, attitudes, and practices related to treatment and prevention of cholera, Haiti, 2010. Emerg Infect Dis. 2011;17(11):2158–61. Epub 2011/12/30. 10.3201/eid1711.110818 22204033 PMC3310585

[82] Steele A, Clarke B, Watkins O. Impact of jerry can disinfection in a camp environment—experiences in an IDP camp in Northern Uganda. J Water Health. 2008;6(4):559–64. 10.2166/wh.2008.072 . 18401121

[83] Guévart E, Van Hecke, C., Noeske J., Sollé J., Bita Fouda, A., Manga B. Diffuseur artisanal de chlore pour désinfecter les puits lors de l'épidémie de choléra de Douala (2004). / [Handmade devices for continuous delivery of hypochlorite for well disinfection during the cholera outbreak in Douala, Cameroon (2004)]. Med Trop (Mars). 2008;68(5):507–13. 19068985

[84] Garandeau R, Trevett A., Bastable A. Cholrination of hand-dug wells in Monrovia. Waterlines. 2006;24(3):19–21.

[85] Einarsdottir J, Passa A, Gunnlaugsson G. Health education and cholera in rural Guinea-bissau. Int J Infect Dis. 2001;5(3):133–8. 10.1016/s1201-9712(01)90087-6 . 11724669

[86] Dunston C, McAfee D, Kaiser R, Rakotoarison D, Rambeloson L, Hoang AT, et al Collaboration, cholera, and cyclones: a project to improve point-of-use water quality in Madagascar. Am J Public Health. 2001;91(10):1574–6. 10.2105/ajph.91.10.1574 11574309 PMC1446828

[87] Quick RE, Venczel LV, Gonzalez O, Mintz ED, Highsmith AK, Espada A, et al Narrow-mouthed water storage vessels and in situ chlorination in a Bolivian community: a simple method to improve drinking water quality. Am J Trop Med Hyg. 1996;54(5):511–6. Epub 1996/05/01. 10.4269/ajtmh.1996.54.511 . 8644907

[88] Quick RE, Gerber ML, Palacios AM, Beingolea L, Vargas R, Mujica O, et al Using a knowledge, attitudes and practices survey to supplement findings of an outbreak investigation: cholera prevention measures during the 1991 epidemic in Peru. Int J Epidemiol. 1996;25(4):872–8. 10.1093/ije/25.4.872 . 8921469

[89] Mahadik VJ, Mbomena J. Impact of health education programme on knowledge, attitude and practice (KAP) of people in cholera affected areas of Luapula Province—Zambia. Med J Zambia. 1983;17(2):32–8. . 6678090

[90] D’Mello-Guyett L, Yates T, Bastable A, Dahab M, Deola C, Dorea C, et al Setting priorities for humanitarian water, sanitation and hygiene research: a meeting report. Conflict and Health. 2018;12(1). 10.1186/s13031-018-0159-8

[91] Grimshaw JM, Russell IT. Effect of clinical guidelines on medical practice: a systematic review of rigorous evaluations. Lancet. 1993;342(8883):1317–22. Epub 1993/11/27. 10.1016/0140-6736(93)92244-n . 7901634

[92] Djulbegovic B, Guyatt GH. Progress in evidence-based medicine: a quarter century on. Lancet. 2017;390(10092):415–23. Epub 2017/02/22. 10.1016/S0140-6736(16)31592-6 . 28215660

[93] WHO. WHO Handbook for Guideline Development. Geneva, Switzerland: 2014.

[94] Tricco AC, Lillie E, Zarin W, O'Brien KK, Colquhoun H, Levac D, et al PRISMA Extension for Scoping Reviews (PRISMA-ScR): Checklist and Explanation. Ann Intern Med. 2018;169(7):467–73. Epub 2018/09/05. 10.7326/M18-0850 . 30178033

[95] Dangour AD, Watson L, Cumming O, Boisson S, Che Y, Velleman Y, et al Interventions to improve water quality and supply, sanitation and hygiene practices, and their effects on the nutritional status of children. Cochrane Database Syst Rev. 2013;(8):CD009382 10.1002/14651858.CD009382.pub2 . 23904195 PMC11608819

[96] Piper JD, Chandna J, Allen E, Linkman K, Cumming O, Prendergast AJ, et al Water, sanitation and hygiene (WASH) interventions: effects on child development in low- and middle-income countries. Cochrane Db Syst Rev. 2017 10.1002/14651858.Cd012613

[97] Fleiss JL, Nee JM, Paik MC. Statistical methods for rates and proportions. 3rd Edition ed New York, USA: Wiley; 2003.

[98] Landis JR, Koch GG. The measurement of observer agreement for categorical data. Biometrics. 1977;33(1):159–74. Epub 1977/03/01. . 843571

[99] Mukandavire Z, Liao S, Wang J, Gaff H, Smith DL, Morris JG Jr. Estimating the reproductive numbers for the 2008–2009 cholera outbreaks in Zimbabwe. Proc Natl Acad Sci U S A. 2011;108(21):8767–72. Epub 2011/04/27. 10.1073/pnas.1019712108 21518855 PMC3102413

[100] MSF. Management of a Cholera Epidemic. Médecins Sans Frontières, 2017.

[101] Oxfam. Cholera Outbreak Guidelines: Preparedness, Prevention and Control. Oxford, UK: Oxfam, 2012.

[102] ACF. Manuel Pratique: Eau, Assainissement, Hygiène dans la Lutte Contre le Choléra. Paris, France: Action Contre la Faim, 2013.

[103] Sphere. The Sphere Project: Humanitarian Charter and Minimum Standards in Humanitarian Response. Geneva, Switzerland: 2018.

[104] UNICEF. Cholera Toolkit. New York, USA: United Nations Children's Fund, 2013.

[105] WHO. Cholera Outbreak, Assessing the Outbreak Response and Improving Preparedness. Geneva, Switzerland: World Health Organisation, 2004.

[106] Global Task Force on Cholera Control. Cholera Outbreak Response: Field Manual (January 2019 Prepress Copy). Geneva, Switzerland: WHO, 2019.

[107] ICDDR'B. COTS Program 2.0. Dhaka, Bangladesh: 2018.

[108] The Lancet. Cholera: ending a 50-year pandemic. The Lancet. 2017;390. 10.1016/S0140-6736(17)32592-8 29131781

[109] Phelps M, Perner ML, Pitzer VE, Andreasen V, Jensen PKM, Simonsen L. Cholera Epidemics of the Past Offer New Insights Into an Old Enemy. J Infect Dis. 2018;217(4):641–9. Epub 2017/11/23. 10.1093/infdis/jix602 29165706 PMC5853221

[110] Yates T, Vujcic JA, Joseph ML, Gallandat K, Lantagne D. Water, sanitation, and hygiene interventions in outbreak response: a synthesis of evidence. Waterlines. 2018;37(1):5–30. 10.3362/1756-3488.17-00015

[111] Wolf J, Hunter PR, Freeman MC, Cumming O, Clasen T, Bartram J, et al Impact of Drinking Water, Sanitation and Hand Washing with Soap on Childhood Diarrhoeal Disease: Updated Meta-Analysis and -Regression. Trop Med Int Health. 2018 Epub 2018/03/15. 10.1111/tmi.13051 . 29537671

[112] Esteves Mills J, Cumming O. The impact of water, sanitation and hygiene on key health and social outcomes: review of evidence. London, UK: SHARE & LSHTM, 2016.

[113] Saif-Ur-Rahman KM, Parvin T, Bhuyian SI, Zohura F, Begum F, Rashid MU, et al Promotion of Cholera Awareness Among Households of Cholera Patients: A Randomized Controlled Trial of the Cholera-Hospital-Based-Intervention-for-7 Days (CHoBI7) Intervention. Am J Trop Med Hyg. 2016;95(6):1292–8. 10.4269/ajtmh.16-0378 27799644 PMC5154442

[114] Merrell DS, Butler SM, Qadri F, Dolganov NA, Alam A, Cohen MB, et al Host-induced epidemic spread of the cholera bacterium. Nature. 2002;417(6889):642–5. 10.1038/nature00778 12050664 PMC2776822

[115] Greenland K, Chipungu J, Curtis V, Schmidt WP, Siwale Z, Mudenda M, et al Multiple behaviour change intervention for diarrhoea control in Lusaka, Zambia: a cluster randomised trial. Lancet Glob Health. 2016;4(12):e966–e77. Epub 2016/11/20. 10.1016/S2214-109X(16)30262-5 . 27855872

[116] Tidwell JB, Gopalakrishnan A, Lovelady S, Sheth E, Unni A, Wright R, et al Effect of Two Complementary Mass-Scale Media Interventions on Handwashing with Soap among Mothers. J Health Commun. 2019;24(2):203–15. Epub 2019/03/27. 10.1080/10810730.2019.1593554 . 30912707

[117] Hirai M, Graham JP, Mattson KD, Kelsey A, Mukherji S, Cronin AA. Exploring Determinants of Handwashing with Soap in Indonesia: A Quantitative Analysis. Int J Environ Res Public Health. 2016;13(9). Epub 2016/09/07. 10.3390/ijerph13090868 27598178 PMC5036701

[118] Rabbi SE, Dey NC. Exploring the gap between hand washing knowledge and practices in Bangladesh: a cross-sectional comparative study. BMC Public Health. 2013;13:89 Epub 2013/02/01. 10.1186/1471-2458-13-89 23363772 PMC3564897

[119] Biran A, Schmidt WP, Wright R, Jones T, Seshadri M, Isaac P, et al The effect of a soap promotion and hygiene education campaign on handwashing behaviour in rural India: a cluster randomised trial. Trop Med Int Health. 2009;14(10):1303–14. Epub 2009/08/28. 10.1111/j.1365-3156.2009.02373.x . 19708896

[120] Freeman MC, Stocks ME, Cumming O, Jeandron A, Higgins JP, Wolf J, et al Hygiene and health: systematic review of handwashing practices worldwide and update of health effects. Trop Med Int Health. 2014;19(8):906–16. 10.1111/tmi.12339 . 24889816

[121] George CM, Zohura F, Teman A, Thomas E, Hasan T, Rana S, et al Formative research for the design of a scalable water, sanitation, and hygiene mobile health program: CHoBI7 mobile health program. BMC Public Health. 2019;19(1):1028 Epub 2019/08/02. 10.1186/s12889-019-7144-z 31366398 PMC6670164

[122] George CM, Sack DA. Integration of water, sanitation and hygiene intervention delivery at health facilities with a reactive ring vaccination programme to reduce cholera. Int J Epidemiol. 2017;46(6):2093–4. Epub 2017/03/25. 10.1093/ije/dyx025 . 28338776 PMC6251573

[123] Roskosky M, Acharya B, Shakya G, Karki K, Sekine K, Bajracharya D, et al Feasibility of a Comprehensive Targeted Cholera Intervention in The Kathmandu Valley, Nepal. Am J Trop Med Hyg. 2019;100(5):1088–97. Epub 2019/03/20. 10.4269/ajtmh.18-0863 30887946 PMC6493959

[124] Parker AA, Stephenson R, Riley PL, Ombeki S, Komolleh C, Sibley L, et al Sustained high levels of stored drinking water treatment and retention of hand-washing knowledge in rural Kenyan households following a clinic-based intervention. Epidemiol Infect. 2006;134(5):1029–36. Epub 2006/01/28. 10.1017/S0950268806005954 16438747 PMC2870483

[125] Briere EC, Ryman TK, Cartwright E, Russo ET, Wannemuehler KA, Nygren BL, et al Impact of integration of hygiene kit distribution with routine immunizations on infant vaccine coverage and water treatment and handwashing practices of Kenyan mothers. J Infect Dis. 2012;205 Suppl 1:S56–64. Epub 2012/02/15. 10.1093/infdis/jir779 . 22315387

[126] Kern E, Verguet S, Yuhas K, Odhiambo FH, Kahn JG, Walson J. Provision of bednets and water filters to delay HIV-1 progression: cost-effectiveness analysis of a Kenyan multisite study. Trop Med Int Health. 2013;18(8):916–24. Epub 2013/05/11. 10.1111/tmi.12127 . 23659539

[127] Azman AS, Luquero FJ, Salje H, Naibei Mbaibardoum N, Adalbert N, Ali M, et al Micro-hotspots of Risk in Urban Cholera Epidemics. J Infect Dis. 2018 Epub 2018/05/15. 10.1093/infdis/jiy283 . 29757428 PMC6107744

[128] Bwire G, Ali M, Sack DA, Nakinsige A, Naigaga M, Debes AK, et al Identifying cholera "hotspots" in Uganda: An analysis of cholera surveillance data from 2011 to 2016. PLoS Negl Trop Dis. 2017;11(12):e0006118 Epub 2017/12/29. 10.1371/journal.pntd.0006118 29284003 PMC5746206

[129] Bwire G, Debes AK, Orach CG, Kagirita A, Ram M, Komakech H, et al Environmental Surveillance of Vibrio cholerae O1/O139 in the Five African Great Lakes and Other Major Surface Water Sources in Uganda. Front Microbiol. 2018;9:1560 Epub 2018/08/21. 10.3389/fmicb.2018.01560 30123189 PMC6085420

[130] Rebaudet S, Sudre B, Faucher B, Piarroux R. Environmental determinants of cholera outbreaks in inland Africa: a systematic review of main transmission foci and propagation routes. J Infect Dis. 2013;208 Suppl 1:S46–54. 10.1093/infdis/jit195 . 24101645

[131] Nelson EJ, Harris JB, Morris JG Jr., Calderwood SB, Camilli A. Cholera transmission: the host, pathogen and bacteriophage dynamic. Nat Rev Microbiol. 2009;7(10):693–702. 10.1038/nrmicro2204 19756008 PMC3842031

[132] Luby SP, Davis J, Brown RR, Gorelick SM, Wong THF. Broad approaches to cholera control in Asia: Water, sanitation and handwashing. Vaccine. 2019 Epub 2019/08/07. 10.1016/j.vaccine.2019.07.084 . 31383486

[133] Garn JV, Sclar GD, Freeman MC, Penakalapati G, Alexander KT, Brooks P, et al The impact of sanitation interventions on latrine coverage and latrine use: A systematic review and meta-analysis. Int J Hyg Environ Health. 2017;220(2 Pt B):329–40. Epub 2016/11/09. 10.1016/j.ijheh.2016.10.001 27825597 PMC5414716

[134] Tamason CC, Bessias S, Villada A, Tulsiani SM, Ensink JH, Gurley ES, et al Measuring domestic water use: a systematic review of methodologies that measure unmetered water use in low-income settings. Trop Med Int Health. 2016 10.1111/tmi.12769 . 27573762

[135] Stelmach RD, Clasen T. Household water quantity and health: a systematic review. Int J Environ Res Public Health. 2015;12(6):5954–74. 10.3390/ijerph120605954 26030467 PMC4483681

[136] De Buck E, Borra V, De Weerdt E, Vande Veegaete A, Vandekerckhove P. A systematic review of the amount of water per person per day needed to prevent morbidity and mortality in (post-)disaster settings. PLoS ONE. 2015;10(5). 10.1371/journal.pone.0126395 25961720 PMC4427459

[137] Pickering AJ, Davis J. Freshwater availability and water fetching distance affect child health in sub-Saharan Africa. Environ Sci Technol. 2012;46(4):2391–7. 10.1021/es203177v . 22242546

[138] Paneth N. Assessing the contributions of John Snow to epidemiology: 150 years after removal of the broad street pump handle. Epidemiology. 2004;15(5):514–6. . 15308944 10.1097/01.ede.0000135915.94799.00

[139] WHO. Guidelines for drinking water quality, 4th edition, incorportating the 1st addendum2017. 631 p.

[140] Jeandron A, Saidi JM, Kapama A, Burhole M, Birembano F, Vandevelde T, et al Water supply interruptions and suspected cholera incidence: a time-series regression in the Democratic Republic of the Congo. PLoS Med. 2015;12(10):e1001893 Epub 2015/10/28. 10.1371/journal.pmed.1001893 26506001 PMC4624412

[141] White G, Bradley W, White A. Drawers of Water: Domestic water use in East Africa. Chicago, USA: The University of Chicago Press, Chicago; 1972.

[142] UN. Sustainable Development Goals: UN; 2015 [cited 2016 02 Sept 2016]. Available from: https://sustainabledevelopment.un.org/?menu=1300.

[143] Nygren BL, Blackstock AJ, Mintz ED. Cholera at the crossroads: the association between endemic cholera and national access to improved water sources and sanitation. Am J Trop Med Hyg. 2014;91(5):1023–8. Epub 2014/09/10. 10.4269/ajtmh.14-0331 25200265 PMC4228869

[144] WHO/UNICEF. Sustainable Development Goals- Goal 6: Ensure availability and sustainable management of water and sanitation for all Geneva, Switzerland2018 [13/05/2019]. Available from: https://unstats.un.org/sdgs/report/2018/goal-06/.

[145] WHO/UNICEF. Progress on Sanitation and Drinking Water: 2015 Update and MDG Assessment. Geneva, Switzerland: World Health Organization & United Nations Children's Fund, 2015.

[146] JMP. WHO/UNICEF Joint Monitoring Programme for Water Supply, Sanitation and Hygiene 2015. Available from: https://washdata.org/monitoring.

[147] Colwell RR. Global climate and infectious disease: the cholera paradigm. Science. 1996;274(5295):2025–31. 10.1126/science.274.5295.2025 . 8953025

[148] Lipp EK, Huq A, Colwell RR. Effects of global climate on infectious disease: the cholera model. Clin Microbiol Rev. 2002;15(4):757–70. Epub 2002/10/05. 10.1128/CMR.15.4.757-770.2002 12364378 PMC126864

[149] Stoltzfus JD, Carter JY, Akpinar-Elci M, Matu M, Kimotho V, Giganti MJ, et al Interaction between climatic, environmental, and demographic factors on cholera outbreaks in Kenya. Infect Dis Poverty. 2014;3(1):37 10.1186/2049-9957-3-37 25328678 PMC4200235

[150] Petticrew M, Knai C, Thomas J, Rehfuess EA, Noyes J, Gerhardus A, et al Implications of a complexity perspective for systematic reviews and guideline development in health decision making. BMJ Glob Health. 2019;4(Suppl 1):e000899 Epub 2019/02/19. 10.1136/bmjgh-2018-000899 30775017 PMC6350708

[151] Guyatt GH, Oxman AD, Vist GE, Kunz R, Falck-Ytter Y, Alonso-Coello P, et al GRADE: an emerging consensus on rating quality of evidence and strength of recommendations. BMJ. 2008;336(7650):924–6. Epub 2008/04/26. 10.1136/bmj.39489.470347.AD 18436948 PMC2335261

[152] Schunemann HJ, Oxman AD, Brozek J, Glasziou P, Bossuyt P, Chang S, et al GRADE: assessing the quality of evidence for diagnostic recommendations. Evid Based Med. 2008;13(6):162–3. Epub 2008/12/02. 10.1136/ebm.13.6.162-a . 19043023

[153] Flemming K, Booth A, Garside R, Tuncalp O, Noyes J. Qualitative evidence synthesis for complex interventions and guideline development: clarification of the purpose, designs and relevant methods. BMJ Glob Health. 2019;4(Suppl 1):e000882 Epub 2019/02/19. 10.1136/bmjgh-2018-000882 30775015 PMC6350756

[154] Higgins JPT, Lopez-Lopez JA, Becker BJ, Davies SR, Dawson S, Grimshaw JM, et al Synthesising quantitative evidence in systematic reviews of complex health interventions. BMJ Glob Health. 2019;4(Suppl 1):e000858 Epub 2019/02/19. 10.1136/bmjgh-2018-000858 30775014 PMC6350707

