# Supplementary material for: Prevention and control of cholera with household and community water, sanitation and hygiene (WASH) interventions: A scoping review of current international guidelines
Source: PLoS One. 2020 Jan 8;15(1):e0226549. doi: 10.1371/journal.pone.0226549 (PMC6948749; doi:10.1371/journal.pone.0226549)
Supplement: S1 Table — (DOCX) [file pone.0226549.s001.docx]

| **Table S1. List of 95 water, sanitation and hygiene (WASH) recommendations featured in eight guidelines for cholera prevention and control** | | | | | | | | | | | | |
| --- | --- | --- | --- | --- | --- | --- | --- | --- | --- | --- | --- | --- |
| **#** | **Recommendation** | **WHO, 2004** | **OXFAM, 2012** | **ACF, 2013** | **UNICEF, 2013** | **MSF, 2017** | **SPHERE, 2018** | **ICDDR'B, 2018** | **GTFCC, 2019** | **Total number of recommendations featured (including when not recommended)** | **Transmission level** | **Theoretical interruption of cholera transmission pathway** |
| **Improving the access to water sources and/or quantity of water** | | | | | | | | | | | | |
| 1 | Assessment and mapping of existing water sources (i.e. availability, types, access, quantity of water, risks of contamination) | ✓ | ✓ | ✓ | ✓ | ✓ | ✓ | ✓ | ✓ | **8** | **Household/ Community** | **Both** |
| 2 | Minimum requirement of 15-20 litres per person per day | ✓ | × | × | ✓ | ✓ | ✓ | ✓ | × | **5** | **Household** | **Human-to-human** |
| 3 | Minimum 500m distance to water sources required, with no more than 15-30 minutes queuing times | × | × | × | × | ✓ | ✓ | ✓ | × | **3** | **Community** | **Environment-to-human** |
| 4 | Installation or repair of temporary or permanent improved water sources (e.g. boreholes, protected wells, protected hand pumps, protected springs, water tankers, water distribution systems including taps to households or public spaces and/or protection of the water source) | ✓ | ✓ | ✓ | ✓ | ✓ | ✓ | × | ✓ | **7** | **Household/ Community** | **Environment-to-human** |
| 5 | Trucking/transport of water where there is no water supply nearby or existing | × | ✓ | ✓ | ✓ | ✓ | ✓ | × | × | **5** | **Community** | **Environment-to-human** |
| 6 | Closing of contaminated or high-risk water points and providing alternatives | × | × | ✓ | ✓ | ✓ | × | × | × | **3** | **Community** | **Environment-to-human** |
| 7 | Installation of bulk water storage at the community-level | × | × | × | ✓ | × | × | × | × | **1** | **Community** | **Environment-to-human** |
| 8 | Monitoring of water quantity at the household-level (e.g. checking sufficient quantity of water at per capita per day) | × | ✓ | ✓ | × | × | ✓ | ✓ | × | **4** | **Household** | **Human-to-human** |
| 9 | Monitoring of water supply at the community-level (e.g. checking water vendors, water tankers and distribution systems) | × | × | ✓ | × | × | ✓ | × | × | **2** | **Community** | **Environment-to-human** |
| **Improving the quality of water: water treatment at source** | | | | | | | | | | | | |
| 10 | A free residual chlorine (FRC) concentration of >0.5 mg/l measured, at source | ✓ | ✓ | ✓ | ✓ | ✓ | ✓ | ✓ | ✓ | **8** | **Community** | **Environment-to-human** |
| 11 | A turbidity less than 5 NTU at the water source, up to 20 NTU acceptable | × | × | ✓ | × | ✓ | ✓ | ✓ | × | **4** | **Community** | **Environment-to-human** |
| 12 | Optimal pH range of water for chlorine to be effective is 6.5-8.5 at point of delivery | × | × | ✓ | × | ✓ | × | × | ✓ | **3** | **Community** | **Environment-to-human** |
| 13 | Water quality tests meeting minimum of <10 CFU/100ml at source, in absence of chlorination | × | × | × | × | × | ✓ | × | × | **1** | **Community** | **Environment-to-human** |
| 14 | Target a higher FRC at pH >7-8, at source | × | × | ✓ | × | ✓ | × | × | × | **2** | **Community** | **Environment-to-human** |
| 15 | Highly turbid water, at source, should not be chlorinated and filtration, coagulation-flocculation or other pre-treatments should be used to reduce turbidity before treatment | ✓ | ✓ | ✓ | × | ✓ | ✓ | ✓ | ✓ | **7** | **Community** | **Environment-to-human** |
| 16 | Use of double dosage of chlorine temporarily for highly turbid water, at source | × | × | × | × | × | ✓ | × | × | **1** | **Community** | **Environment-to-human** |
| 17 | Microbiological testing for *Vibrio cholerae* at source | × | × | ✓ | × | × | × | × | × | **1** | **Community** | **Environment-to-human** |
| 18 | Monitoring of water quality at source | × | ✓ | ✓ | ✓ | × | × | ✓ | ✓ | **5** | **Community** | **Environment-to-human** |
| 19 | Bulk or batch chlorination of water sources (e.g. in-line chlorination of water distribution systems, temporary bladders, water tanks and trucking), with dosage determined by jar tests | ✓ | ✓ | ✓ | ✓ | ✓ | ✓ | ✓ | × | **7** | **Community** | **Environment-to-human** |
| 20 | Bucket chlorination at water sources of household containers (5, 10 or 20 litre jerry cans) with an effective chlorine residual (0.2 to 0.5mg/litre), with dosage determined by jar tests | × | ✓ | ✓ | ✓ | ✓ | × | × | × | **4** | **Community** | **Environment-to-human** |
| 21 | Chlorination of unimproved water sources (e.g. unprotected wells, unlined wells) | × | × | × | NR | NR | × | × | × | **2NR** | **Community** | **Environment-to-human** |
| **Improving the quality of water: point of use (POU) and safe storage** | | | | | | | | | | | | |
| 22 | A free residual chlorine (FRC) concentration of 0.2 to 0.5mg/l measured after 30 minutes contact time measured, at the point of use | × | ✓ | ✓ | ✓ | × | × | ✓ | ✓ | **5** | **Household** | **Human-to-human** |
| 23 | A turbidity less than 5 NTU, at point of use | × | × | × | × | × | ✓ | ✓ | × | **2** | **Household** | **Human-to-human** |
| 24 | Target a higher FRC at pH >7-8, at point of use | × | × | ✓ | × | ✓ | × | × | × | **2** | **Household** | **Human-to-human** |
| 25 | Highly turbid water, at point of use, should not be chlorinated and filtration, coagulation-flocculation or other pre-treatments should be used to reduce turbidity before treatment | ✓ | ✓ | ✓ | × | ✓ | ✓ | ✓ | ✓ | **7** | **Household** | **Human-to-human** |
| 26 | Use of double dosage of chlorine temporarily for highly turbid water, at point of use | × | × | × | × | × | ✓ | × | × | **1** | **Household** | **Human-to-human** |
| 27 | Microbiological testing for *Vibrio cholerae* at point of use | × | × | ✓ | × | × | × | × | × | **1** | **Household** | **Human-to-human** |
| 28 | Monitoring of water quality at the household level | × | ✓ | ✓ | ✓ | ✓ | ✓ | ✓ | ✓ | **7** | **Household** | **Human-to-human** |
| 29 | Distribution of household water treatment products/technologies | × | ✓ | ✓ | ✓ | ✓ | ✓ | ✓ | ✓ | **7** | **Household** | **Human-to-human** |
| 30 | Promotion of household water treatment products/technologies | ✓ | ✓ | ✓ | ✓ | ✓ | ✓ | ✓ | ✓ | **8** | **Household** | **Human-to-human** |
| 31 | Distribution of safe water storage containers | × | × | ✓ | ✓ | ✓ | ✓ | × | ✓ | **5** | **Household** | **Human-to-human** |
| 32 | Promotion of cleaning, coverage and/disinfection of safe water storage containers | ✓ | ✓ | ✓ | ✓ | ✓ | ✓ | × | ✓ | **7** | **Household** | **Human-to-human** |
| **Improving the access to and use of sanitation facilities and reducing exposure to faeces** | | | | | | | | | | | | |
| 33 | Assessment and mapping of existing sanitation facilities (i.e. coverage, types, access, risks of contamination) | ✓ | ✓ | × | ✓ | ✓ | ✓ | × | ✓ | **6** | **Community** | **Environment-to-human** |
| 34 | Sanitation facilities should be a minimum 50m distance to sanitation facilities including facilities that are: >30m away from a groundwater source, 1.5-2m above the water table, limit vector breeding (e.g. flies and mosquitoes), private, considerate of gender, safe to use and have an adequate water supply | × | × | × | ✓ | ✓ | ✓ | ✓ | ✓ | **5** | **Community** | **Environment-to-human** |
| 35 | Limit or control open defecation | × | × | ✓ | ✓ | × | ✓ | × | ✓ | **4** | **Community** | **Environment-to-human** |
| 36 | Installation or repair of household sanitation | ✓ | ✓ | × | ✓ | ✓ | × | × | ✓ | **5** | **Community** | **Environment-to-human** |
| 37 | Distribution of latrine construction materials to households | × | × | × | × | × | ✓ | ✓ | × | **2** | **Community** | **Environment-to-human** |
| 38 | Installation or repair of communal latrines (e.g. in marketplaces, harbours, schools, refugee camps) | × | ✓ | ✓ | ✓ | ✓ | ✓ | ✓ | × | **6** | **Community** | **Environment-to-human** |
| 39 | Distribution of potties, scoops or nappies to dispose of child faeces | × | × | × | × | × | ✓ | × | × | **1** | **Household/ Community** | **Both** |
| 40 | Promotion of latrine construction and use (e.g. behaviour change communication (BCC), Community Led Total Sanitation (CLTS), social marketing) | ✓ | × | × | ✓ | × | ✓ | × | ✓ | **4** | **Community** | **Environment-to-human** |
| 41 | Replacement of bucket latrines, public or shared latrines or trenches as soon as possible, and/or provision of these options in emergency contexts | ✓ | × | × | × | ✓ | ✓ | × | × | **3** | **Community** | **Environment-to-human** |
| 42 | Establish collection, transport and disposal of plastic bag-based sanitation, if used or introduced | × | × | × | ✓ | × | ✓ | × | × | **2** | **Community** | **Environment-to-human** |
| 43 | Promotion of sharing latrines in urban settings | × | × | × | ✓ | × | × | × | × | **1** | **Community** | **Environment-to-human** |
| 44 | Promotion of faeces burial ("cat method") | × | × | ✓ | ✓ | × | × | × | × | **2** | **Community** | **Environment-to-human** |
| 45 | Distribution of latrine cleaning materials for communal and/or household latrines (e.g. detergent, lime, etc.) | × | ✓ | × | ✓ | ✓ | ✓ | × | × | **4** | **Community** | **Environment-to-human** |
| **Behaviour change interventions to improve personal, domestic and food hygiene practices** | | | | | | | | | | | | |
| 46 | Promotion of handwashing after defecation, before eating, before preparing food, before feeding a child, after cleaning a child's faeces and after contact with a cholera case | ✓ | ✓ | ✓ | ✓ | ✓ | ✓ | ✓ | ✓ | **8** | **Household** | **Human-to-human** |
| 47 | Promotion of safe water collection, treatment and storage (e.g. for drinking and cooking) | ✓ | ✓ | ✓ | ✓ | ✓ | ✓ | × | ✓ | **7** | **Household** | **Human-to-human** |
| 48 | Promotion of safe food preparation, cooking and storage (e.g. covering food to avoid flies and contamination, promotion of breastfeeding) | ✓ | ✓ | × | ✓ | ✓ | ✓ | ✓ | ✓ | **7** | **Household** | **Human-to-human** |
| 49 | Promotion of safe dish washing after eating | ✓ | × | × | ✓ | × | × | × | × | **2** | **Household** | **Human-to-human** |
| 50 | Promotion of safe defecation practices (e.g. no open defecation, use of latrines, cleaning of latrines, safe disposal of child faeces) | ✓ | ✓ | × | ✓ | ✓ | ✓ | ✓ | ✓ | **7** | **Household/ Community** | **Both** |
| 51 | Promotion of solid waste disposal | × | ✓ | ✓ | ✓ | × | ✓ | × | × | **4** | **Household/ Community** | **Both** |
| 52 | Promotion of safe food preparation to street food vendors and restaurants | × | × | × | ✓ | × | × | × | × | **1** | **Community** | **Human-to-human** |
| 53 | Assessment and analysis of hygiene practices (i.e. identify high risk practices, cultural practices and preferences) | × | × | × | × | × | ✓ | × | × | **1** | **Household/ Community** | **Human-to-human** |
| 54 | Hygiene promotion through house-to-house visits or community meetings | × | ✓ | ✓ | ✓ | ✓ | ✓ | ✓ | ✓ | **7** | **Household/ Community** | **Human-to-human** |
| 55 | Hygiene promotion and cholera awareness using mass media (e.g. radio, television, SMS, social media) | ✓ | ✓ | ✓ | ✓ | ✓ | ✓ | ✓ | ✓ | **8** | **Household/ Community** | **Human-to-human** |
| 56 | Distribution of hygiene promotion materials (e.g. Information Education Communication (IEC)) | × | ✓ | ✓ | ✓ | × | × | ✓ | ✓ | **5** | **Household** | **Human-to-human** |
| 57 | Hygiene promotion in schools and other institutions (e.g. churches, mosques) | × | ✓ | ✓ | ✓ | × | ✓ | × | × | **4** | **Community** | **Human-to-human** |
| 58 | Hygiene promotion targeted at funerals, marriages, religious festivals and other public gatherings | ✓ | ✓ | ✓ | ✓ | × | × | × | ✓ | **5** | **Household/ Community** | **Human-to-human** |
| 59 | Hygiene promotion including handwashing and solid waste management among food and water vendors and marketplaces | × | × | ✓ | ✓ | ✓ | × | × | ✓ | **4** | **Household/ Community** | **Human-to-human** |
| 60 | Monitoring of food safety among food vendors and marketplaces including closures, enforcement of food hygiene standards with public health authorities | × | ✓ | ✓ | ✓ | × | × | ✓ | ✓ | **5** | **Household/ Community** | **Human-to-human** |
| 61 | Hygiene and health promotion through behaviour change communication (BCC), social marketing or community engagement (CE) or other theory-based techniques and frameworks | × | ✓ | ✓ | ✓ | × | ✓ | ✓ | ✓ | **6** | **Household/ Community** | **Human-to-human** |
| 62 | Promotion of alternative food-based solutions to limit cholera transmission (e.g. use acidifying foods such as lime, tomatoes, yoghurt) | ✓ | × | × | ✓ | × | × | × | × | **2** | **Household** | **Human-to-human** |
| 63 | Monitoring of hygiene items and practices (e.g. soap use, changes to hygiene practices) | × | ✓ | ✓ | ✓ | ✓ | ✓ | × | ✓ | **6** | **Household** | **Human-to-human** |
| **Distribution of hygiene materials or non-food items (NFIs)** | | | | | | | | | | | | |
| 64 | Distribution of soap to households | × | ✓ | ✓ | ✓ | ✓ | ✓ | ✓ | ✓ | **7** | **Household** | **Human-to-human** |
| 65 | Distribution of soap at the community level | × | ✓ | × | ✓ | ✓ | ✓ | × | × | **4** | **Household/ Community** | **Human-to-human** |
| 66 | Distribution of hygiene kits which include soap, hand washing devices, water treatment products, water storage containers and/or cholera IEC materials | × | ✓ | ✓ | ✓ | ✓ | ✓ | × | × | **5** | **Household** | **Human-to-human** |
| 67 | Distribution of hygiene kits with materials sufficient for 1 month | × | ✓ | ✓ | × | × | × | × | × | **2** | **Household** | **Human-to-human** |
| 68 | Distribution of drinking cups, washing bowls and eating equipment to persons in refugee camps, prisons and other institutions | × | × | × | ✓ | × | × | × | × | **1** | **Household** | **Human-to-human** |
| 69 | Distribution of detergents for cleaning water storage containers | × | ✓ | × | × | × | × | × | × | **1** | **Household** | **Human-to-human** |
| 70 | Distribution of items to schools and other communal facilities to aid personal hygiene, food preparation and waste management | × | × | × | ✓ | × | ✓ | × | × | **2** | **Household** | **Human-to-human** |
| 71 | Installation of handwashing points in public places (e.g. markets, schools, public toilets) | × | ✓ | ✓ | ✓ | ✓ | ✓ | ✓ | ✓ | **7** | **Household/ Community** | **Human-to-human** |
| **Promotion or distribution of disinfection and cleaning of households, community spaces and/or materials** | | | | | | | | | | | | |
| 72 | Promotion of household cleaning and/or disinfection (e.g. floors, furniture and surfaces) | × | × | × | ✓ | ✓ | × | × | × | **2** | **Household** | **Human-to-human** |
| 73 | Disinfection of households with chlorine spraying (especially vomit and faeces) | × | NR | NR | NR | NR | × | × | × | **4NR** | **Household** | **Human-to-human** |
| 74 | Provision of disinfection materials to households for household cleaning and disinfection (e.g. detergents, 0.5-2% chlorine solution) | × | NR | ✓ | ✓ | ✓ | × | × | × | **3/1NR** | **Household** | **Human-to-human** |
| 75 | Promotion of safe laundry practices, including disinfection of clothes and bedding of cholera cases with chlorine, boiling for 5 minutes or drying in the sun; alternatively burn or bury with the deceased | ✓ | × | ✓ | ✓ | ✓ | ✓ | ✓ | ✓ | **7** | **Household** | **Human-to-human** |
| 76 | Disinfection of household items that cannot be washed in the sun (e.g. mattresses) | × | × | × | × | ✓ | × | × | × | **1** | **Household** | **Human-to-human** |
| 77 | Disinfection of non-households with chlorine spraying (e.g. in vehicles, marketplaces) | × | NR | NR | NR | NR | × | × | × | **3NR** | **Community** | **Human-to-human** |
| 78 | Wash vehicles that have been used to transport cholera cases | × | × | × | ✓ | × | × | × | × | **1** | **Household/ Community** | **Human-to-human** |
|  | | | | | | | | | | | | |
| **Improving dead body management and safe funeral practices** | | | | | | | | | | | | |
| 79 | Promotion of safe and hygienic practices for corpse preparation and discouraging funeral feasts (e.g. through house-to-house visits, community meetings, and through community and/or religious leaders) | ✓ | ✓ | × | ✓ | ✓ | × | ✓ | ✓ | **6** | **Household/ Community** | **Human-to-human** |
| 80 | Engage with local authorities, community and/or religious leaders for safe funeral practices and corpse management | × | ✓ | ✓ | ✓ | ✓ | × | ✓ | ✓ | **6** | **Household/ Community** | **Both** |
| 81 | Encouragement of funerals within 24hours of a death or as soon as possible | × | × | × | × | ✓ | × | × | ✓ | **2** | **Household** | **Human-to-human** |
| 82 | Promotion or provision of hygiene materials to households for safe and hygienic corpse preparation (e.g. detergents, 0.5-2% chlorine solution, body bags) | ✓ | NR | ✓ | ✓ | ✓ | × | × | × | **4/1NR** | **Household** | **Human-to-human** |
| 83 | Promotion or provision of safe burial sites (e.g. away from water sources) | × | ✓ | ✓ | ✓ | ✓ | × | × | ✓ | **5** | **Community** | **Environment-to-human** |
| 84 | Adaptation or discouragement of funeral feasts and community meetings | × | × | × | ✓ | ✓ | × | ✓ | ✓ | **4** | **Household/ Community** | **Human-to-human** |
| 85 | Disinfection of corpses with chlorine, and fill mouth and anus with cotton wool soaked in chlorine | ✓ | ✓ | ✓ | ✓ | ✓ | × | ✓ | ✓ | **7** | **Household/ Community** | **Both** |
| 86 | Provision of handwashing facilities at funerals | × | × | ✓ | ✓ | × | × | × | ✓ | **3** | **Household/ Community** | **Human-to-human** |
| 87 | Allocation of designated health workers to supervise hygienic practices at funerals | ✓ | × | ✓ | × | ✓ | × | ✓ | ✓ | **5** | **Household/ Community** | **Human-to-human** |
| 88 | Disinfection of graves (e.g. using lime) | × | ✓ | × | × | × | × | × | × | **1** | **Community** | **Environment-to-human** |
| **Improving the management of wastewater and faecal sludge** | | | | | | | | | | | | |
| 89 | Installation or maintenance of wastewater drains or on-site drainage, particularly around water sources | × | ✓ | ✓ | ✓ | × | ✓ | × | × | **4** | **Community** | **Environment-to-human** |
| 90 | Treatment of faecal sludge in latrines (e.g. with chlorinated lime or lime) | × | ✓ | × | × | × | × | × | × | **1** | **Community** | **Environment-to-human** |
| 91 | Adequate measures are in place for latrine desludging, handling, transportation and disposal (if off-site), including avoidance of latrine desludging | × | × | × | × | × | ✓ | × | ✓ | **2** | **Community** | **Environment-to-human** |
| **Provision of interventions that improve solid waste disposal** | | | | | | | | | | | | |
| 92 | Assessment and mapping of solid waste disposal and hazards (i.e. define hazards, identify adequate measures) | × | × | × | × | × | ✓ | × | × | **1** | **Community** | **Environment-to-human** |
| 93 | Encouragement of or support to safely manage solid waste control in markets, harbours and other human environments where solid waste presents a health hazard | × | ✓ | × | ✓ | × | ✓ | × | × | **3** | **Community** | **Environment-to-human** |
| 94 | Organisation of periodic solid waste campaigns and/or a system to periodically remove waste from waste zones | × | × | × | ✓ | × | ✓ | × | × | **2** | **Community** | **Environment-to-human** |
| **Use of vector control interventions to reduce flies** | | | | | | | | | | | | |
| 95 | Reduction of fly populations through insecticide spraying in breeding areas | × | NR | × | × | × | × | × | × | **1NR** | **Community** | **Environment-to-human** |
| NR- Not Recommended by a guideline; WHO- World Health Organization, MSF- Médecins Sans Frontières, ICDDR’B- International Centre for Diarrhoeal Disease Research Bangladesh, ACF- Action Contre la Faim, UNICEF- United Nations Children’s Fund, GTFCC- Global Task Force on Cholera Control | | | | | | | | | | | | |
