# Supplementary material for: Prevention and control of cholera with household and community water, sanitation and hygiene (WASH) interventions: A scoping review of current international guidelines
Source: PLoS One. 2020 Jan 8;15(1):e0226549. doi: 10.1371/journal.pone.0226549 (PMC6948749; doi:10.1371/journal.pone.0226549)
Supplement: S1 Appendix — (DOCX) [file pone.0226549.s002.docx]

| Appendix S1 Search Strategy and resources searched | |
| --- | --- |
| Organisation | **Website** |
| Global WASH Cluster | www.washcluster.org |
| World Health Organization (WHO) | [www.who.int](http://www.who.int) |
| United Nations Children’s Fund (UNICEF) | [www.unicef.org](http://www.unicef.org) |
| United Nations High Commissioner for Refugees (UNHCR) | www.unhcr.org |
| United Nations Office for the Coordination of Humanitarian Affairs (UNOCHA) | www.unocha.org |
| World Food Programme (WFP) | [www.wfp.org](http://www.wfp.org) |
| International Organization for Migration (IOM) | www.iom.org |
| Médecins Sans Frontières (MSF) | [www.msf.org](http://www.msf.org) |
| Oxfam | [www.oxfam.org.uk](http://www.oxfam.org.uk) |
| International Red Cross and Red Crescent (ICRC) | [www.icrc.org](http://www.icrc.org) |
| International Federation of the Red Cross (IFRC) | [www.ifrc.org](http://www.ifrc.org) |
| Action Contre la Faim (ACF) | www.actionagainsthunger.org |
| International Rescue Committee (IRC) | www.irc.org |
| Care International | www.careinternational.org.uk |
| Save the Children | www.nrc.no |
| Norwegian Refugee Council (NRC) | www.savethechildren.org.uk |
| The Sphere Project | www.sphereproject.org |
| US Centers for Disease Control and Prevention (CDC) | www.cdc.gov |
| International Centre for Diarrhoeal Disease Research Bangladesh (ICDDR’B) | www.icddrb.org |
| London School of Hygiene and Tropical Medicine (LSHTM) | www.lshtm.ac.uk |
| Water, Education and Development Centre (WEDC) | [www.wedc.lboro.ac.uk](http://www.wedc.lboro.ac.uk) |
| Relief Web | [www.reliefweb.int](http://www.reliefweb.int) |
| Humanitarian Response | www.humanitarianresponse.info |
