# Supplementary material for: Prevention and control of cholera with household and community water, sanitation and hygiene (WASH) interventions: A scoping review of current international guidelines
Source: PLoS One. 2020 Jan 8;15(1):e0226549. doi: 10.1371/journal.pone.0226549 (PMC6948749; doi:10.1371/journal.pone.0226549)
Supplement: S2 Appendix — (DOCX) [file pone.0226549.s003.docx]

| Appendix S2 Search Terms |
| --- |
| Water Quality |
| (water adj3 (treatment or quality or cleaning or microbiology))  OR  (water adj3 (purif* or chlor* or decontamination or filt* or disinfect* or floccul* or radiat* or irradiati* or sediment*))  OR  (water adj3 (storage or recontamination or re-contamination))  OR  (water adj3 (drinking or consumption)) |
| Water Supply |
| (water adj3 (supply or availability or access or connect* or distance or improve* or distribut* or quantity or volume or piped or standpipe$1 or handpump$1)) |
| Sanitation |
| (toilet* or latrine* or pit or pits or sanita* or ecosan or "ecological sanita*" or privy or WC or "water closet")  OR  ((f$eces or f$ecal or excre* or waste or defecation) adj3 (disposal or manag* or service*))  OR  (sewage or sewer$1 or sewerage)  OR  "septic tank$"  OR  "open defecation" |
| Hygiene |
| (hygiene or handwashing or hand-washing or (hand$1 adj3 wash*) or (hand$1 adj3 hygien*) or (hand$1 adj3 clean) or (hand$1 adj3 disinfect*) or (hand$1 adj3 sterili*) or soap*) |
| Cholera |
| (cholera or cholera*)  OR  (vibrio cholerae or V. cholerae or vibrio)  OR  (diarrhoea or (diarrh*) adj3 (acute or watery or rice or water or loose or bloody))  OR  (stool adj3 (acute or watery or rice or water or loose or bloody))  OR  Dysentery adj3  (acute or watery or rice or water or loose or bloody)) |
| Guidelines |
| (guidelines or guide)  OR  (manual*)  OR  (protocol*) |
| Prevention and control |
| (prevention OR control OR implementation OR delivery OR management OR practice OR response OR programme OR program) |
