# Supplementary material for: Prevention and control of cholera with household and community water, sanitation and hygiene (WASH) interventions: A scoping review of current international guidelines
Source: PLoS One. 2020 Jan 8;15(1):e0226549. doi: 10.1371/journal.pone.0226549 (PMC6948749; doi:10.1371/journal.pone.0226549)
Supplement: S3 Appendix — (DOCX) [file pone.0226549.s004.docx]

| Appendix S3 Excluded guidelines | | | |
| --- | --- | --- | --- |
| Organisation | **Year** | **Title** | **Reason for exclusion** |
| WaterAid | 2017 | The War To End Cholera: How A Lack Of Clean Water And Sanitation Are Contributing To The Global Spread Of Disease | Document is not a guideline |
| Global Task Force on Cholera Control | 2017 | Ending Cholera: A Global Roadmap To 2030 | Document is not a guideline |
| ACF | 2017 | WASH ‘Nutrition: A Practical Guidebook On Increasing Nutritional Impact Through Integration Of WASH And Nutrition Programmes (For Practitioners In Humanitarian And Development Contexts) | Guideline is not specific to cholera prevention and control |
| WHO | 2017 | Guidelines For Drinking Water Quality 4^th^ Edition, Incorporating The 1st Addendum | Guideline is not specific to cholera prevention and control |
| MSF | 2016 | Evidence-Based Guidelines For Centralizes Chlorination In Emergencies: Background To The New FRC Guidance: Study Methodologies And Outcomes | Guideline is not specific to cholera prevention and control |
| WHO | 2016 | Health Care Without Avoidable Infections: The Critical Role Of Infection Prevention And Control | Guideline for WASH in Health Care Facilities and/or Infection Prevention and Control |
| Oxfam | 2013 | Oxfam Minimum Requirements For WASH Programmes | Guideline is not specific to cholera prevention and control |
| LSHTM | 2013 | Choose Soap Toolkit | Guideline is not specific to cholera prevention and control |
| ICRC | 2013 | Water, Sanitation, Hygiene And Habitat In Prisons | Guideline is not specific to cholera prevention and control |
| Sphere | 2011 | The Sphere Project: Humanitarian Charter And Minimum Standards in Humanitarian Response | Historical version of guideline |
| Oxfam | 2011 | Oxfam Guidelines For Water Treatment In Emergencies | Guideline is not specific to cholera prevention and control |
| MSF | 2010 | Public Health Engineering In Precarious Situations | Guideline is not specific to cholera prevention and control |
| WHO | 2009 | Who Guidelines On Hand Hygiene In Health Care | Guideline for WASH in Health Care Facilities and/or Infection Prevention and Control |
| UNICEF | 2009 | Water, Sanitation And Hygiene (WASH) Cluster Coordination Handbook: A Practical Guide For All Those Involved In The Water, Sanitation And Hygiene Cluster | Guideline is not specific to cholera prevention and control |
| WHO | 2009 | Core Components For Infection Prevention And Control Programmes | Guideline for WASH in Health Care Facilities and/or Infection Prevention and Control |
| UNHCR | 2008 | Guidance For UNHCR Field Operations On Water And Sanitation Services | Guideline is not specific to cholera prevention and control |
| IFRC | 2008 | Household Water Treatment And Safe Storage In Emergencies | Guideline is not specific to cholera prevention and control |
| Heymann, D. L | 2008 | Control Of Communicable Diseases Manual | Guideline is not specific to cholera prevention and control |
| WHO | 2008 | Essential Environmental Health Standards In Health Care | Guideline for WASH in Health Care Facilities and/or Infection Prevention and Control |
| ICDDR’B | 2006 | Cots Programme | Historical version of guideline |
| ACF | 2006 | Water, Sanitation And Hygiene For Populations At Risk | Guideline is not specific to cholera prevention and control |
| WHO | 2005 | Communicable Disease Control In Emergencies | Guideline is not specific to cholera prevention and control |

| Organisation | Year | Title | Reason for exclusion |
| --- | --- | --- | --- |
| Cairncross, S. Feachem, R. | 2005 | Environmental Health Engineering In The Tropics: An Introductory Text | Guideline is not specific to cholera prevention and control |
| World Bank | 2005 | The Handwashing Handbook: A Guide For Developing A Hygiene Promotion Programme | Guideline is not specific to cholera prevention and control |
| MSF | 2004 | Cholera Guidelines | Historical version of guideline |
| Sphere | 2004 | The Sphere Project: Humanitarian Charter And Minimum Standards in Humanitarian Response | Historical version of guideline |
| Young, H; Borrel, A; Holland, D; Salama, P. | 2004 | Public Nutrition In Complex Emergencies | Guideline is not specific to cholera prevention and control |
| House, S.; Reed, B. | 2004 | Emergency Water Sources: A Guideline For Selection And Treatment | Guideline is not specific to cholera prevention and control |
| WHO | 2002 | Environmental Health In Emergencies And Disasters | Guideline is not specific to cholera prevention and control |
| Harvey, P.; Baghri, S; Reed, B. | 2002 | Emergency Sanitation Assessment And Programme Design | Guideline is not specific to cholera prevention and control |
| Davis, J; Lambert, R. | 2002 | Engineering In Emergencies: A Practical Guide For Relief Workers | Guideline is not specific to cholera prevention and control |
| Sphere | 2000 | The Sphere Project: Humanitarian Charter And Minimum Standards in Humanitarian Response | Historical version of guideline |
| Ferron, S.; Morgon, J.; O’Reilly, M. | 2000 | Hygiene Promotion: A Practical Manual For Relief And Development | Guideline is not specific to cholera prevention and control |
